# Occurrence of Total and Proteinase K-Resistant Alpha-Synuclein in Glioblastoma Cells Depends on mTOR Activity

**DOI:** 10.3390/cancers14061382

**Published:** 2022-03-08

**Authors:** Larisa Ryskalin, Rosangela Ferese, Gabriele Morucci, Francesca Biagioni, Carla L. Busceti, Fabrizio Michetti, Paola Lenzi, Alessandro Frati, Francesco Fornai

**Affiliations:** 1 Department of Translational Research and New Technologies in Medicine and Surgery, University of Pisa, Via Roma 55, 56126 Pisa, Italy; larisa.ryskalin@unipi.it (L.R.); gabriele.morucci@unipi.it (G.M.); paola.lenzi@unipi.it (P.L.); 2 Istituto di Ricovero e Cura a Carattere Scientifico (I.R.C.C.S.) Neuromed, Via Atinense 18, 86077 Pozzilli, Italy; rosangela.ferese@neuromed.it (R.F.); francesca.biagioni@neuromed.it (F.B.); carla.busceti@neuromed.it (C.L.B.); alessandro.frati@uniroma1.it (A.F.); 3 Departmentof Neuroscience, Università Cattolica del Sacro Cuore, Largo Francesco Vito 1, 00168 Rome, Italy; fabrizio.michetti@unicatt.it; 4 IRCCS (Istituto di Ricovero e Cura a Carattere Scientifico) San Raffaele Scientific Institute, Università Vita-Salute San Raffaele, Via Olgettina 6, 20121 Milan, Italy; 5 Neurosurgery Division, Human Neurosciences Department, Sapienza University, 00135 Roma, Italy

**Keywords:** glioblastoma multiforme, synucleins, cell-clearing systems, autophagy vacuoles, mechanistic target of rapamycin, transmission electron microscopy, qRT-PCR, siRNA

## Abstract

**Simple Summary:**

The accumulation of alpha-synuclein (α-syn) is considered a pathological hallmark of the neurodegenerative disorders known as synucleinopathies. The clearance of α-syn depends on autophagy activity, which is inhibited by the mechanistic target of rapamycin (mTOR). Thus, it is likely that α-syn accumulation may occur whenever mTOR is overactive and autophagy is suppressed. In fact, the lack of effective autophagy increases the amount of α-syn and may produce protein aggregation. Therefore, in the present study, we questioned whether cells from glioblastoma multiforme (GBM), a lethal brain neoplasm, wherein mTOR is upregulated and autophagy is suppressed, may overexpress α-syn. In fact, a large quantity of α-syn is measured in GBM cells compared with astrocytes, which includes proteinase K-resistant α-syn. Rapamycin, while inhibiting mTOR activity, significantly reduces the amount of α-syn and allocates α-syn within autophagy-like vacuoles.

**Abstract:**

Alpha-synuclein (α-syn) is a protein considered to be detrimental in a number of degenerative disorders (synucleinopathies) of which α-syn aggregates are considered a pathological hallmark. The clearance of α-syn strongly depends on autophagy, which can be stimulated by inhibiting the mechanistic target of rapamycin (mTOR). Thus, the overexpression of mTOR and severe autophagy suppression may produce α-syn accumulation, including the proteinase K-resistant protein isoform. Glioblastoma multiforme (GBM) is a lethal brain tumor that features mTOR overexpression and severe autophagy inhibition. Cell pathology in GBM is reminiscent of a fast, progressive degenerative disorder. Therefore, the present work questions whether, as is analogous to neurons during degenerative disorders, an overexpression of α-syn occurs within GBM cells. A high amount of α-syn was documented in GBM cells via real-time PCR (RT-PCR), Western blotting, immunohistochemistry, immuno-fluorescence, and ultrastructural stoichiometry, compared with the amount of β- and γ-synucleins and compared with the amount of α-syn counted within astrocytes. The present study indicates that (i) α-syn is overexpressed in GBM cells, (ii) α-syn expression includes a proteinase-K resistant isoform, (iii) α-syn is dispersed from autophagy-like vacuoles to the cytosol, (iv) α-syn overexpression and cytosol dispersion are mitigated by rapamycin, and (v) the α-syn-related GBM-like phenotype is mitigated by silencing the SNCA gene.

## 1. Introduction

Alpha-synuclein (α-syn) has been comprehensively investigated over the past few decades due to its occurrence in various neurodegenerative disorders. These studies stem from the original report showing that a point mutation in the α-syn gene (SNCA) leads to inherited degeneration that recapitulates the pathology of Parkinsonism, thus posing the first case of a Mendelian-like transmitted Parkinsonism [1]. Further studies documented that a large amount of α-syn, even when expressed with a normal structure, produces widespread neurodegeneration [2]. The accumulation of α-syn within specific neurons and/or glial cells occurs in some degenerative disorders that are known as synucleinopathies [3,4,5,6,7]. It is remarkable that, when α-syn accumulation extends from the neurons to the glia, multiple-system atrophy occurs, which is characterized by a fast-progressing degeneration with a short survival time [3,4,5,6,7,8,9,10,11,12,13]. This suggests that the accumulation of α-syn within glia accelerates disease progression and spreads the pathology to numerous brain areas.

Robust evidence indicates that the clearance of α-syn depends on autophagy, which is inhibited by the mechanistic target of rapamycin (mTOR) [14]. Thus, it is likely that α-syn accumulation may be a common phenomenon occurring in a wide range of disorders where mTOR is overactive and autophagy is suppressed.

This is likely to happen in disorders featuring mTOR upregulation since this type of molecular complex acts as an autophagy suppressor. It is well known that glioblastoma multiforme (GBM), a lethal brain tumor, features mTOR overactivation, which is concomitant with autophagy suppression [15,16,17,18]. The impairment of autophagy fosters the initiation and progression of GBM. In fact, the amount of autophagy suppression in GBM cells is related to tumor malignancy. This includes infiltration, lethality, stemness, spreading, invasiveness, and relapse [19,20,21,22]. Since autophagy has a prominent role in removing α-syn, it is crucial to investigate the occurrence of α-syn in GBM cells.

Thus, in the present study, we questioned whether cells from GBM may overexpress both native and proteinase K (PK)-resistant α-syn. This study compares the number of primary transcripts of α-syn with those of β-syn and γ-syn and quantifies the occurrence and subcellular placement of the protein α-syn in GBM cells compared with astrocytes. The specific role of α-syn in modulating the GBM phenotype is documented by reducing the synthesis of α-syn using specific siRNA. The mTOR inhibitor and autophagy inducer, rapamycin, was administered to establish whether the amount of total α-syn, the amount of PK-resistant α-syn, and the misplacement of α-syn were all reduced in GBM cells.

## 2. Materials and Methods

### 2.1. Cell Cultures

The GBM cell line U87MG (Cell Bank, IRCC San Martino-IST, Genova, Italy) was grown in DMEM (Sigma Aldrich, Milan, Italy) with 1% MEM + nonessential amino acids (MEM-NEAA), 10% fetal bovine serum (FBS), and 100 μg of streptomycin and penicillin (50 IU/mL) (Sigma Aldrich). Cells were maintained at 37 °C with 5% CO_2_.

Normal human fetal-derived astrocytes (NHA; Lonza, Basel, Switzerland, CC-2565; lot #0000514417) were grown according to the manufacturer’s specifications (AGM BulletKit, Lonza, Euroclone) and kept at 37 °C with 5% CO_2_. The cell medium was renewed twice a week. Astrocytes were cultured to reach roughly 80% confluence, then subcultures were obtained after partial digestion with ReagentPack™ subculturing reagents (Lonza; CC-5034).

In order to carry out cytochemistry, immuno-cytochemistry, and immuno-fluorescence experiments under light microscopy, 5 × 10^4^ cells were seeded on coverslips within 24-well plates at a final volume of 1 mL/well.

For electron microscopy, qRT-PCR, and Western blots, 1 × 10^6^ cells were seeded in 6-well plates at a final volume of 2 mL/well.

### 2.2. Cell Treatments

Cells were treated with increasing doses of rapamycin (1 nM, 10 nM, 100 nM, and 1000 nM) in 0.01% DMSO for 24 h, in order to produce the inhibition of mTOR. Control cells were maintained within a growth medium containing 0.01% DMSO.

The dosages used here include the range of 10–100 nM needed for therapeutic response, based on rapamycin acting as an mTOR inhibitor. The doses and times of rapamycin exposure include those used in previous studies [23,24,25,26,27]. These doses modulate the expression of mRNA transcripts from a number of genes that are key to the fate of GBM cells. These encompassed genes relate to stemness and neuronal differentiation [23], as well as those involved in mitochondrial turnover [24,25].

### 2.3. Proteinase K Treatment

To reveal the occurrence of nondigested α-syn, both the control and rapamycin-treated cells were exposed to the denaturing agent, proteinase K (PK) (Sigma Aldrich). PK treatment consisted of incubation with PK aqueous solution containing 400 μg/mL of PK for 1 min at 37 °C. This protocol was applied before beginning the immunostaining procedures (namely, immuno-peroxidase and immuno-electron microscopy), as previously described [23,28,29,30,31,32]. For the immunoblotting, samples were pretreated with PK aqueous solution for 20 min at 37 °C.

As demonstrated in a previous study [23], such a slight digestion by PK does not affect the sample morphology or protein antigenicity, thus allowing researchers to detect and evaluate the distribution, at a subcellular level, of frankly detectable PK-resistant α-syn.

The outcome PK pretreatment was evaluated by sodium dodecyl sulfate (SDS)-PAGE. Proteins (40 μg) from control and PK-treated samples were dissolved and then separated by electrophoresis (12% SDS polyacrylamide gel). After electrophoresis, the pattern of protein bands was visualized directly on a stain-free enabled Bio-Rad UV trans-illuminator (ChemiDoc^TM^ MP, Bio-Rad Laboratories). A ChemiDoc System (Bio-Rad Laboratories) was used for image analysis.

### 2.4. Silencing RNA Transfection

Small silencing RNAs targeting the α-syn gene were obtained from Ambion (Life Technologies, Carlsbad, CA, USA). The siRNA sequences used were as follows: sense, GAAUAAUGACGUAUUGUGAtt (5′ > 3′); antisense, UCACAAUACGUCAUUAUUCtt (5′ > 3′).

As a negative control (scramble RNA, scRNA), Silencer^®^ Select Negative Control #1 siRNA (catalog #4390843, Life Technologies, Monza, Italy, Bio-Rad laboratories, Segrate (MI), Italy) was used.

U87MG cells were seeded onto multi-well plates as follows:

(i) For the Western blot, cells were seeded onto 6-well plates, 1 × 10^6^ cells per well;
(ii) For light microscopy, cells were seeded onto 24-well plates, 2 × 10^5^ cells per well.

Transfection was carried out when the cell confluence reached roughly 70%.

Briefly, lipofectamine RNAiMAX reagent (Thermo Fisher Scientific, San Francisco, CA, USA) was used for transfection, according to the manufacturer’s instructions. Accordingly, 25 pmol and 5 pmol siRNA/scRNA solutions, dissolved in Opti-MEM medium (Thermo Fisher Scientific), were prepared in order to transfect 6- and 24-well plates, respectively, at 37 °C in the presence of 5% CO_2_ for 24 h.

The silencing efficiency of α-syn was evaluated by Western blot. The effects of α-syn silencing on cell survival and cell phenotype were evaluated under light microscopy using the trypan blue (TB) assay, hematoxylin and eosin (H&E) staining, and immuno-peroxidase against the cancer stem-cell markers nestin and CD133.

### 2.5. Trypan Blue Assay

To assess the percentage of dying cells, at the end of α-syn silencing, 25 μL cell suspensions from non-transfected cells and cells transfected with α-syn siRNA or scRNA were added to 37.5 μL of PBS containing 1% TB. After incubation for 5 min at room temperature, 10 μL of this solution was used to perform cell counting using a Brüker glass chamber. Values were expressed as the mean percentage ± SEM of TB clearly positive cells among the total cells, which values were obtained from three independent experiments.

### 2.6. Hematoxylin and Eosin (H&E) Staining

U87MG cells (corresponding to non-transfected cells and cells transfected with α-syn siRNA or scRNA) were fixed in a 4% paraformaldehyde phosphate-buffered solution (PBS) for 15 min, subsequently washed in PBS, and immersed in hematoxylin solution (Sigma Aldrich) for about 20 min. Hematoxylin staining was stopped by washing the slides under running water. Afterward, cells were plunged within the eosin solution for a few minutes (Sigma Aldrich). After repeated washing to remove the excess dye, cells were dehydrated in increasing alcohol solutions, clarified in xylene, covered with DPX mounting medium (Sigma Aldrich), and finally observed under a Nikon Eclipse 80i light microscope (Nikon, Tokyo, Japan), equipped with a fluorescent lamp and a digital camera connected to the NIS Elements software for image analysis.

Cell counting was carried out under light microscopy at 20× magnification within two distinct slides for each experimental group. Only distinct, nonoverlapped cells were considered for the counts. The number of stained cells was expressed as the mean percentage ± SEM of the control group (i.e., non-transfected cells). Numbers were obtained from three independent experiments.

### 2.7. Hoechst Staining

U87MG cells were seeded at a density of 5 × 10^4^ on coverslips within 24-well plates in a final volume of 1 mL/well.

Apoptotic nuclear morphology was determined using Hoechst 33342 DNA staining, according to the manufacturer’s protocol (Invitrogen, Waltham, MA, USA). Briefly, 5 μg/mL of Hoechst 33342 was added to viable cells for 30 min in the dark at 37 °C and immediately visualized using a Nikon Eclipse 80i light microscope (Nikon, Tokyo, Japan), under a fluorescent lamp. The counting of apoptotic cells was carried out under light microscopy at 20× magnification, within two distinct slides for each experimental group. The number of apoptotic cells was expressed as the mean percentage ± SEM of clearly apoptotic cells (showing nuclear condensation) among the total cells, which were obtained from three independent experiments.

### 2.8. Immuno-Cytochemistry under Light Microscopy

After treatments, the culture medium was removed. Cells were fixed with a solution containing 4% paraformaldehyde in PBS for 15 min at room temperature, to carry out immuno-fluorescence or immuno-peroxidase staining.

#### 2.8.1. Immuno-Fluorescence

Cells were exposed to Triton X 0.1% for 15 min in PBS and then incubated in a blocking solution (PBS containing 10% normal goat serum) for 1 h at room temperature. Then, cells were incubated overnight at 4 °C with rabbit polyclonal anti-α-syn primary antibody (1:100; Sigma, Sigma Aldrich, St. Luis, MO, USA, Table 1), diluted in a PBS solution containing 2% normal goat serum. After rinsing with PBS, the antigen–antibody reaction was revealed using the anti-rabbit fluorescent secondary antibody Alexa Fluor 488 (1:200; Life Technologies, Carlsbad, CA, USA), at room temperature for 1 h.

The cell nuclei were visualized after cell incubation with the nuclear dye DAPI (Sigma Aldrich) diluted 1:3000 in PBS for 5 min. After rinsing in PBS, cells were cover-slipped with the mounting medium, Fluoroshield (Sigma Aldrich). The Nikon Eclipse 80i light microscope, equipped with a fluorescent lamp and a digital camera connected to the NIS Elements software for image analysis (Nikon, Tokyo, Japan), was used to observe the cell samples. Pictures of DAPI- and α-syn-stained cells were independently acquired and then merged. The final panels were prepared using Adobe Photoshop.

#### 2.8.2. Immuno-Peroxidase

For immuno-peroxidase staining, fixed cells were permeabilized using Triton X 0.1% (Sigma Aldrich) for 15 min in PBS, rinsed in PBS to facilitate antigen retrieval, and then incubated in 3% hydrogen peroxide (H_2_O_2_) for 20 min to block endogenous peroxidase activity, followed by treatment with a blocking solution containing 10% normal goat serum in PBS for 1 h at room temperature. Then, cells were incubated overnight at 4 °C, with the primary antibody diluted in PBS containing 2% normal goat serum. The following primary antibodies were used: mouse monoclonal anti-α-syn antibody (1:1000; Abcam, Cambridge, UK; Table 1) and rabbit polyclonal anti-nestin antibody (1:1000; Millipore, Temecula, CA, USA). The secondary antibody was an anti-mouse or anti-rabbit biotin-conjugated antibody (1:200; Vector Laboratories, Burlingame, CA, USA), and the reaction was carried out for 1 h at room temperature. Cells were exposed to the avidin–biotin complex (ABC, Vector Laboratories) for 1 h. Then, they were exposed for 3 min to the peroxidase substrate diaminobenzidine (DAB, Vector Laboratories), at room temperature. After dehydration in increasing alcohol solutions, cells were clarified in xylene, cover-slipped with DPX mounting medium (Sigma Aldrich), and observed under a Nikon Eclipse 80i light microscope (20× magnification).

### 2.9. Densitometry Analysis under Light Microscopy

Densitometry analysis of the α-syn-immunostained cells, within three distinct microscopic fields, was carried out under light microscopy (40× magnification). In all fields, cells were not overlapping, which allowed the distinguishing of single cells. Image J software was used for measuring densitometry within the nucleus, cytosol, or whole cell. Counts were carried out by two observers, who were blind to the treatments. Data were obtained from *N* = 30 cells per group and are given as the mean percentage ± SEM of optical density for each experimental group (assuming the controls to be 100%).

### 2.10. SDS-PAGE Immunoblotting

Cell samples were homogenized in ice-cold lysis buffer containing phosphatase and protease inhibitors at 4 °C. For protein determinations (Bradford procedure), 1 μL of homogenate was used. Sodium dodecyl sulfate-polyacrylamide gels (12%) were used for protein separation (20 µg). Samples were transferred onto Immuno-PVDF membranes (Bio-Rad, Milan, Italy) for 1 h. Tween-20 Tris-buffered saline (TTBS) (100 mM Tris-HCl, 0.9% NaCl, 1% Tween-20, pH 7.4) containing 5% nonfat dry milk was used to block the filters. Then, membranes were incubated in a solution containing mouse anti-α-syn (1:800; BD Transduction Laboratories, San Jose, CA, USA) or rabbit anti-ribosomal protein pS6 primary antibodies (pS6 RP; 1:1000; Cell Signaling Technology, Danvers, MA, USA) overnight at 4 °C. Incubation with mouse anti-α-syn antibodies (1:800; BD Transduction Laboratories) was also carried out in proteinase K-treated samples (see Section 2.3).

β-Actin was used as a housekeeping protein. Blots were incubated with primary mouse monoclonal anti-β-actin antibody (1:50,000; Sigma Aldrich) for 1 h at room temperature. After washing with TTBS buffer, samples were incubated for 1 h with secondary peroxidase-conjugated antibodies (anti-mouse or anti-rabbit, 1:3000; Calbiochem, Milan, Italy). Enhanced chemiluminescence (GE Healthcare, Milan, Italy) was used to reveal the immunostaining. ImageJ software was used for densitometry analysis. Data are given as the mean ± SEM of the optical density for each experimental group. For proteinase K (PK)-resistant α-syn, quantification of the immune-blotting was carried out according to two approaches: (i) as the ratio between PK-resistant α-syn and β-actin (from analogous PK-untreated samples); (ii) as the ratio between PK-resistant α-syn and monomeric α-syn from PK-untreated samples. Values are given as the mean percentage ± SEM. In fact, the quantification of PK-resistant α-syn using a classic housekeeping approach in the very same PK-treated sample is impossible, due to the digestion of the housekeeping protein. An alternative approach consists of using Triton to extract the amount of housekeeping protein before PK administration. However, variability in protein extraction with Triton ends up biasing the results by adding further experimental variability (protein extraction). The potential to express the amount of PK-resistant α-syn using the reference of total α-syn that was detected in analogous samples was promising. However, due to the potential digestion by PK of various polymers of α-syn, this method provides data that reflect the decrease in oligomers and monomers compared with the polymers of the protein. In fact, PK alters the pattern of α-syn immune-reactive bands in the gel. Thus, the most reliable approach consists of referring α-syn to the housekeeping protein β-actin, already used in non-PK-treated analogous samples where total α-syn is present.

### 2.11. RNA Extraction for qRT-PCR

To further validate α-syn detection through antibody-based procedures, and to specifically assess the gene expression of α-syn compared with β-syn and γ-syn, nonbiased quantitative real-time PCR (qRT-PCR) was carried out.

According to the manufacturer’s instructions, total RNA from cells using TRIzol Reagent (Invitrogen, Life Technologies, Waltham, MA, USA) was isolated, the concentration and purity of RNA samples using Nanodrop 2000 (Thermo Scientific, Life Technologies, Waltham, MA, USA) were determined, and total RNA (100 ng) was reverse-transcribed (RT) with SuperScript^®^ VILO™ (Invitrogen, Life Technologies).

### 2.12. qRT-PCR

A CFX Connect^TM^ Real-Time System (Bio-Rad, Hercules, CA, USA) was used for amplification and detection. The PCR mix contained 10 μL of SYBR Green PCR Master (Applied Biosystems, Foster City, CA, USA), 0.5 μM of each primer, and 0.8 μL of RT reaction mix. The following steps allowed amplifying the PCR mix: 50 °C for 1 min, 95 °C for 10 min, followed by 40 cycles of 95 °C for 30 s and 54 °C for 1 min. The following primers were designed using GenBank (http://www.ncbi.nlm.nih.gov/ (accessed on 13 November 2019)):

- Alpha-synuclein, SNCA (NM_007308.2)
  5′–AGGAGTGGTTCATGGAGTG–3′, 5′–AAGCCAGTGGCAGCAGCT–3′;
- Beta-synuclein, SNCB (NM_003085)
  5′–ATCTGGGAGGAGCTGTGTTC–3′, 5′–CTCTGGCTCATACTCCTGAT–3′;
- Gamma-synuclein, SNCG (NM_003087)
  5′–ACCAAGCAGGGGGTGACGGA– 3′, 5′–GGTGACCGCGATGTTCTCC–3′;
- β-Actin (NM_001101.3)
  5′–GTGCGTGACATTAAGGAG–3′, 5′–GCCAGACAGCACTGTGT–3′;
- β-Globin (NM_000518.4)
  5′–CTAAGGTGAAGGCTCATG–3′, 5′–GATAGGCAGCCTGCACT–3′.

Each run included a positive control (DNA), negative control (distilled water), and RT-negative control (total RNA sample).

The comparative Ct method (also known as the ΔΔCt method) [33] was used to calculate the relative quantification. β-Actin and β-globin were selected as internal references. Ct values are given as the mean values of three PCRs. Two independent experiments were carried out to confirm gene expression, using both β-actin and β-globin as internal references.

### 2.13. Transmission Electron Microscopy

After centrifugation at 1000× *g* for 5 min, the cell pellet was rinsed in PBS and fixed in 2.0% paraformaldehyde and 0.1% glutaraldehyde in 0.1 M PBS (pH 7.4) for 90 min at 4 °C. Then, specimens were post-fixed in 1% OsO_4_ for 1 h at 4 °C, dehydrated in ethanol, and embedded in epoxy resin. At the end of the plain TEM or immuno-cytochemistry procedure, ultrathin slices were counterstained with a saturated solution in distilled water of uranyl acetate and lead citrate; finally, they were examined using a JEOL JEM-100SX transmission electron microscope (JEOL, Tokyo, Japan). For ultrastructural morphometry, grids containing non-serial ultrathin slices (70–90 nm thick) were examined by TEM, at a magnification of 8000×. Several grids were analyzed in order to count a total of 30 cells from each experimental group.

### 2.14. Post-Embedding Immuno-Cytochemistry

In previous studies, the combination of aldehydes, OsO_4_, and epoxy resin for immuno-gold-based ultrastructural morphometry was validated [34,35,36]. Ultrathin slices collected on nickel grids were incubated on droplets of aqueous sodium meta-periodate (NaIO_4_) for 30 min at room temperature, in order to remove OsO_4_ [35].

This step is key for ultrastructural morphometry since it allows the detection, localization, and stoichiometry counts of protein placement within clearly defined subcellular compartments. Gold particles appear as round electron-dense spots, each one corresponding to a single α-syn molecule. This provides the total quantitative assessment and compartmentalization of α-syn. This is routinely applied according to analogous immuno-gold quantification [37,38,39,40].

After this step, the grids were incubated at room temperature in a blocking solution (10% goat serum and 0.2% saponin for 20 min). The grids were then incubated with the primary antibody in PBS containing 0.2% saponin and 1% goat serum in a humidified chamber overnight at 4 °C. The following primary antibodies were used: rabbit anti-α-syn (Sigma Aldrich; Table 1) diluted to 1:50, mouse anti-α-syn (Abcam; Table 1) diluted to 1:50, rabbit anti-LC3 (Abcam) diluted to 1:50, and mouse anti-cathepsin D (Sigma Aldrich) diluted to 1:10. Then, the grids were incubated with the secondary antibodies conjugated with gold particles (10 nm or 20 nm mean diameter, BB International, Treviso (TV), Italy) diluted to 1:20 in PBS containing 0.2% saponin and 1% goat serum for 1 h at room temperature. Control slices were incubated with secondary antibody only. After incubation with 1% glutaraldehyde for 3 min, the grids were washed with distilled water and stained with uranyl acetate and lead citrate.

### 2.15. Ultrastructural Morphometry

Several grids containing non-serial ultrathin slices were examined by TEM (8000× magnification) [41]. This magnification allows researchers to concomitantly identify both single immuno-gold particles and specific cell organelles. The number of immuno-gold particles was counted in 30 cells from each experimental group. This was achieved by examining quite a few grids (each grid containing non-serial slices with at least 10 cells). Within the ultrathin slice, each observer began scanning from one corner of the squared grid, proceeding through equally spaced parallel sweeps for the whole slice. Random selection of the scanned slices reduced bias due to variations of cell density or the intensity of gold labeling in the whole specimen. The assessments of vacuoles and the measurement of immuno-gold particles were carried out according to the method reported by Lenzi et al. (2016) [34,42,43,44].

For each cell, counts included (i) the total number of α-syn immuno-gold particles, (ii) the number of α-syn immuno-gold particles placed within the cytosol, (iii) the number of α-syn immuno-gold particles placed within the nucleus, (iv) the number of α-syn immuno-positive vacuoles, and (v) the number of α-syn immuno-gold particles placed within each vacuole.

Moreover, the numbers of total vacuoles, LC3-positive vacuoles, cathepsin D-positive vacuoles, and double LC3 + α-syn- and cathepsin D + α-syn-positive vacuoles were counted.

The given values express the mean number of immuno-gold particles or positive vacuoles per cell out of 30 cells counted.

### 2.16. Specificity of Anti-α-Syn Primary Antibodies (Specificity of α-Syn Detection in the Study)

In order to validate the specificity of α-syn detection, α-syn immuno-cytochemistry under light microscopy was compared with immuno-cytochemistry under electron microscopy and Western blotting, using various anti-α-syn primary antibodies, as reported in Table 1.

In detail, α-syn staining using a full-length protein antibody (Abcam) was comparable with that obtained using the anti-α-syn Sigma antibody, which detects endogenous levels of total syn protein. Similar data were obtained for BD primary antibodies. This is in line with preliminary studies that confirmed the high degree of similarity among data obtained using these currently available antibodies [45]. The use of the internal control (only a secondary antibody) further indicated the specificity of the data (Supplementary Figure S1). The amount of α-syn compared with β-syn and γ-syn was further validated through the measurement of the primary transcript with RT-PCR. This latter procedure confirmed the results obtained with antibody-based procedures (Western blotting and immunocytochemistry under light and electron microscopy) concerning a higher amount of α-syn in GBM cells compared with astrocytes.

### 2.17. Statistical Analysis

Values related to H&E-stained cells are expressed as the mean percentage ± SEM of cells counted in control (i.e., non-transfected cells). Values related to apoptotic cells (evaluated by Hoechst staining) and TB-positive cells are given as the mean percentage ± SEM of total cells. All these data were obtained from three independent experiments (*N* = 3), each carried out in duplicate. Statistical analysis was carried out on *n* = 6 counts per experimental group.

Values related to the densitometry of immuno-fluorescent and immuno-peroxidase-stained cell cultures are given as the mean percentage ± SEM of optical density for each experimental group (assuming the controls to be 100%). Densitometry data were obtained from three independent experiments (*N* = 3) and refer to a total of *n* = 30 cells per experimental group.

Values related to ultrastructural morphometry are given as the mean ± SEM or the mean percentage ± SEM. Counts were carried out in three independent experiments (*N* = 3) and refer to a total of *n* = 30 cells per experimental group.

For Western blot experiments, values are given as the mean ± SEM of optical density for each experimental group. The immunoblotting of α-syn in astrocytes and GBM cells in the baseline, as well as the subsequent α-syn siRNA transfection densitometry data, refers to *n* = 4 and *n* = 3, respectively, of sample blots per experimental group obtained in *N* = 1 experiment. For the immunoblotting of α-syn in GBM cells treated with rapamycin with or without PK, densitometry data consisting of *n* = 3 values for each group refer to *N* = 3 independent experiments.

Densitometric data for the immunoblotting of pS6 in GBM cells treated with rapamycin, consisting of *n* = 3 values (baseline, 10 nM, and 100 nM) and *n* = 2 values (1000 nM), refer to *N* = 1 independent experiments. The number of independent experiments (*N*) and the total number of immunoblotting samples used for measuring the optical density (*n*) are reported in the related figure legends. The qRT-PCR data were obtained from *N* = 2 independent experiments, each carried out in duplicate. Statistical analysis was carried out in *n* = 4 data for the experimental groups, using statistical software (GraphPad Prism version 6.0, S. Diego, CA, USA).

Inferential statistics to compare groups was carried out using a one-way analysis of variance (ANOVA) with Scheffé’s post hoc or Bonferroni test. The null hypothesis (H_0_) was rejected for *p* < 0.05.

## 3. Results

### 3.1. α-Syn Is Abundant in U87MG Cells

**Figure 1:**
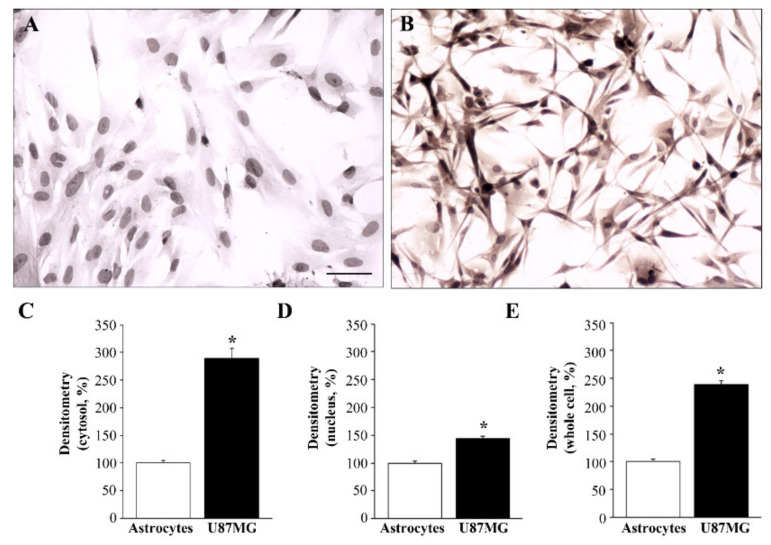
Differential expression of α-syn immuno-cytochemistry between astrocytes and U87MG cells. Representative pictures of α-syn immuno-cytochemistry within astrocytes (**A**) compared with U87MG cells (**B**). Immuno-peroxidase shows that, in baseline conditions, U87MG cells exhibit marked α-syn immunoreactivity in the cytosol, whereas α-syn immunostaining is barely detectable within the cytosol of astrocytes. Graphs report the densitometry of α-syn staining within the cytosol (**C**), nucleus (**D**), and whole cell (**E**) for α-syn staining within astrocytes, compared with GBM cells. Data are given as the mean percentage ± SEM of optical density in each experimental group (assuming the astrocytes to be control = 100% density) obtained from *n* = 30 cells (astrocytes or U87MG cells), in *N* = 3 independent experiments. * *p* < 0.05 compared with astrocytes. Scale bar = 32.5 μm.

As shown in the representative pictures in Figure 1, while α-syn was scarce in astrocytes (Figure 1A), it was found in copious amounts in U87MG cells (Figure 1B, Supplementary Figure S2). The difference in α-syn levels between astrocytes and U87MG cells within the cytosol, nucleus, and the whole cell was measured by densitometry, and the results are reported in the graphs of Figure 1C–E, respectively.

### 3.2. α-Syn Amount and Compartmentalization, Measured by Ultrastructural Stoichiometry

**Figure 2:**
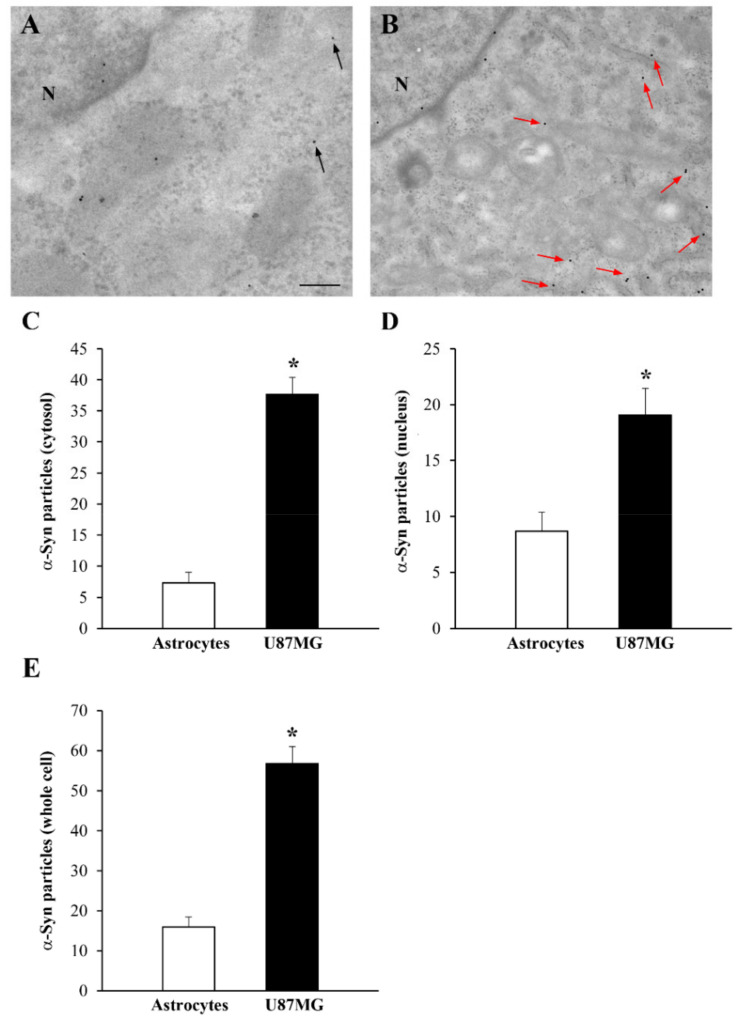
Occurrence and distribution of α-syn within U87MG cells and astrocytes. Representative electron micrographs of α-syn in astrocytes (**A**) and U87MG cells (**B**). In baseline conditions, a few scattered α-syn immuno-gold particles (black arrows) were found within the cytosol of astrocytes. In contrast, in U87MG cells, α-syn immuno-gold particles were abundant (red arrows). The representative distribution suggests that α-syn occurred within vacuoles in the cytosol of astrocytes, while it was uniformly dispersed in the cytosol of U87MG cells. Graphs report the number of α-syn immuno-gold particles within the cytosol (**C**), nucleus (**D**), and whole cell (**E**) within astrocytes, compared with U87MG cells. N = nucleus. Data are given as the mean ± SEM from *n* = 30 cells (astrocytes or U87MG cells) obtained in *N* = 3 independent experiments. * *p* < 0.05 compared with astrocytes. Scale bar = 0.11 μm.

As reported in the representative Figure 2A, α-syn immuno-gold particles were rarely found (black arrows) within the cytosol of astrocytes, in contrast to the representative Figure 2B, where high levels of α-syn (red arrows) were present within the cytosol of U87MG cells. When counting immuno-gold particles within the cytosol, the amount of α-syn exceeded fourfold that counted within astrocytes in U87MG cells (graph of Figure 2C). In the nucleus, the difference was roughly twofold (Figure 2D). Thus, in the whole cell, the amount of α-syn in U87MG cells was three times that of the amount measured within astrocytes, as reported in the graph of Figure 2E.

### 3.3. In U87MG Cells Compared with Astrocytes, Gene Expression of α-Syn Occurs in Excess Compared with β-Syn and γ-Syn

To further validate studies showing a high amount of α-syn in GBM cells using antibody-based procedures, we analyzed α-syn using nonbiased quantitative real-time PCR (qRT-PCR).

To be specific, the ΔΔCt method was used to calculate fold changes in the total expression levels of all three synucleins (α-syn, β-syn, and γ-syn) in baseline conditions within astrocytes and U87MG cells.

**Figure 3:**
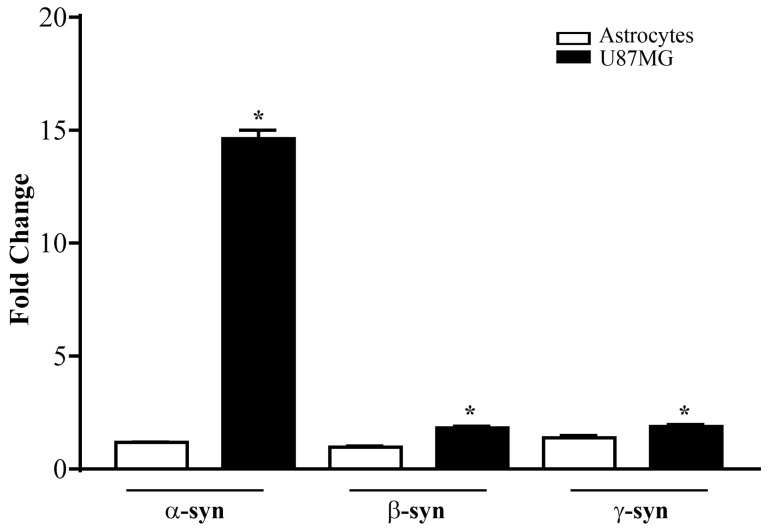
mRNA levels for α-syn, β-syn, and γ-syn in astrocytes and U87MG cells. α-Syn, β-syn, and γ-syn gene transcripts were assessed by quantitative RT-PCR analysis in astrocytes and U87MG cells in baseline conditions. Data are given as the mean ± SEM of *n* = 4 samples from *N* = 2 independent experiments, normalized with two different internal references, globin, and actin. * *p* < 0.05, compared with astrocytes.

As reported in the graph of Figure 3, the transcripts of all synucleins were significantly higher in U87MG cells compared with astrocytes. Remarkably, the α-syn transcript level was substantially in excess (tenfold) compared with astrocytes, while the difference in the expression of β-syn and γ-syn, albeit significantly higher in U87MG cells compared with astrocytes, was negligible compared with α-syn (see the graph in Figure 3).

This approach validated the specificity of antibody-based detection of a high amount of α-syn under light and electron microscopy.

### 3.4. Rapamycin Reduces α-Syn Immunostaining Dose-Dependently

Since U87MG cells are characterized by mTOR-dependent autophagy suppression, while autophagy clears α-syn levels, it is expected that rapamycin, by acting as an mTOR-dependent autophagy activator, dose-dependently mitigates the severe overexpression of α-syn in U87MG cells.

**Figure 4:**
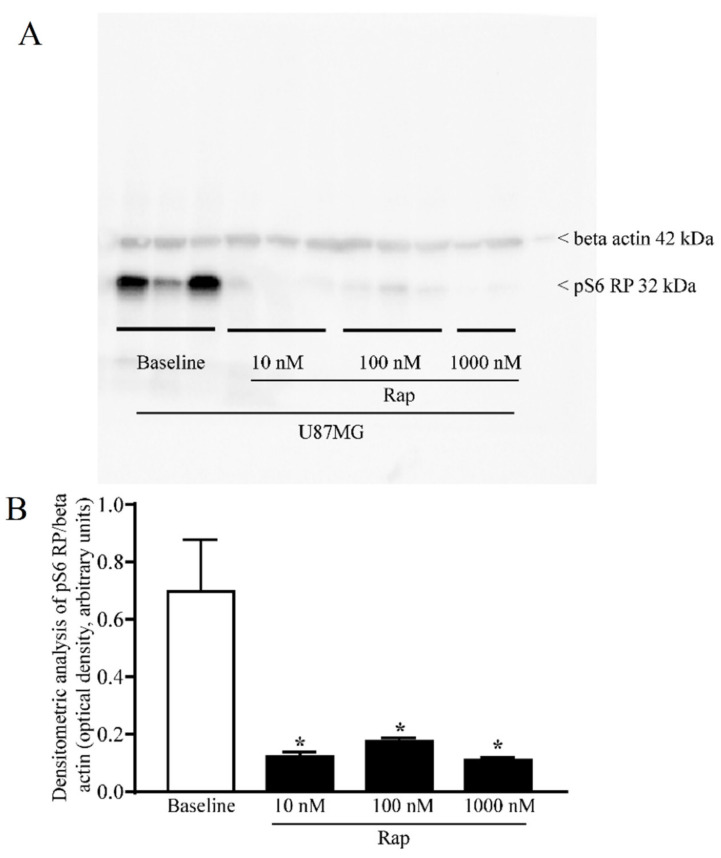
Rapamycin dose-dependently reduced the pS6 ribosomal protein assessed by immunoblotting. (**A**) Representative immunoblotting for the ribosomal protein pS6 (pS6 RP) and the housekeeping protein β-actin in U87MG cells, in baseline conditions and following rapamycin exposure. (**B**) The ratio between the optical densities of pS6RP and β-actin. Data are given as the mean ± SEM of the optical density referring to *n* = 6 (baseline, 10 nM, 100 nM) and *n* = 4 (1000 nM) samples from different cultures obtained in *N* = 2 experiments. * *p* < 0.05 compared with baseline.

**Figure 5:**
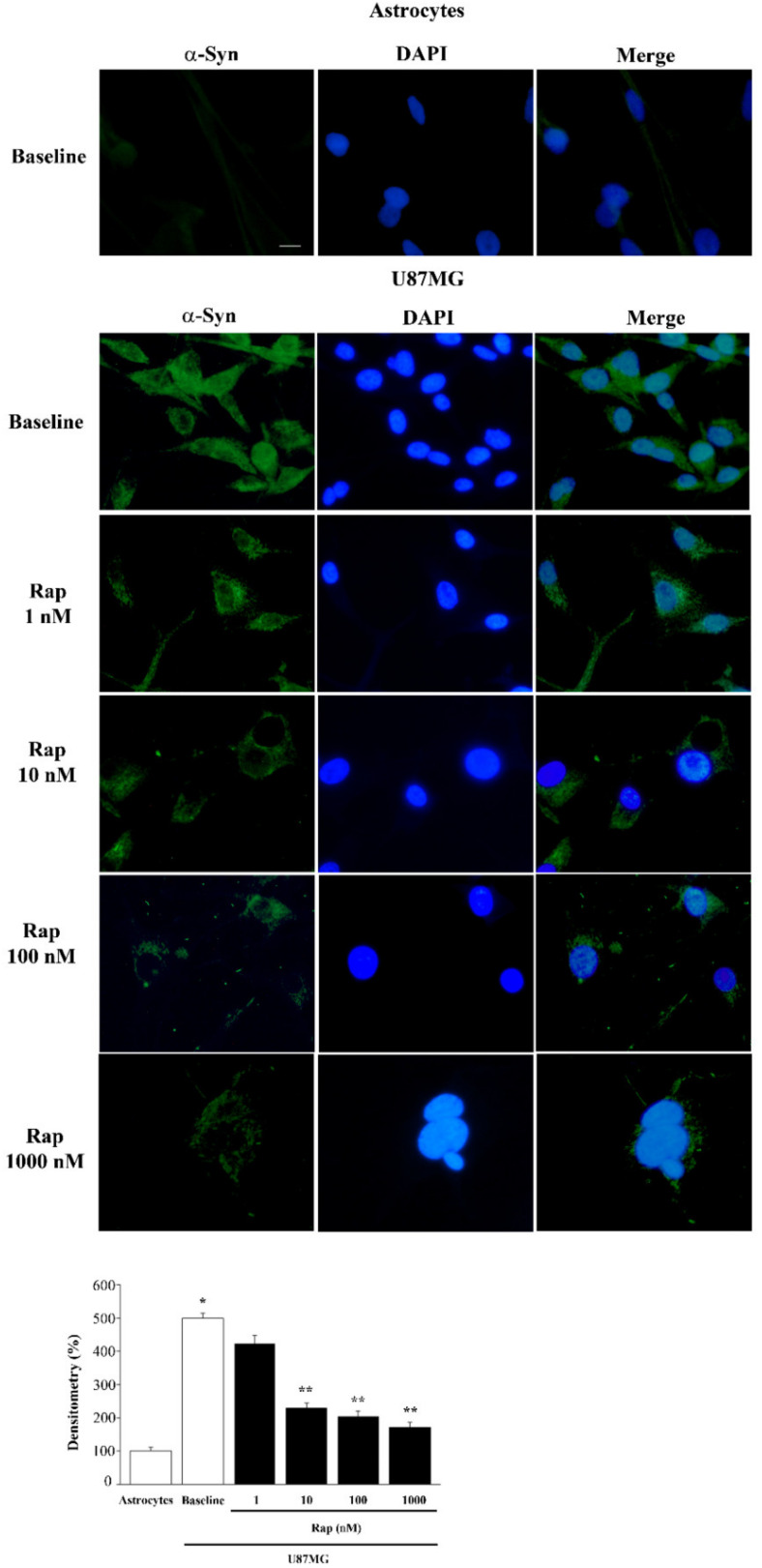
Rapamycin dose-dependently reduced α-syn immuno-fluorescence. Representative pictures of α-syn-positive astrocytes and U87MG cells, in baseline conditions and following rapamycin (Rap) exposure. Each cell nucleus is visualized using DAPI (blue). In these cells, the amount of α-syn is stained by a green fluorescent-labeled secondary antibody (Alexa 488, green). The graph reports the densitometry of α-syn immuno-fluorescence within astrocytes compared with U87MG cells, in baseline conditions and following rapamycin exposure. Data are given as the mean percentage ± SEM of optical density for each experimental group (assuming controls as 100%) obtained from *n* = 30 cells per group in a total of *N* = 3 independent experiments. * *p* < 0.05 compared with astrocytes; ** *p* < 0.05 compared with baseline and Rap 1 nM in U87MG. Scale bar = 12 μm.

A wide range of rapamycin doses was administered in order to firmly inhibit mTOR, as assayed by the downstream mTORC1 product, the ribosomal protein pS6 (representative blots and graph of Figure 4), which confirmed, in the present experimental setting, that rapamycin was effective in blocking mTOR activity. When increasing doses of rapamycin were administered for 24 h to U87MG cells, α-syn levels detected by immuno-fluorescence were suppressed as reported in the representative pictures and graph of Figure 5. The dose-dependent reduction in α-syn staining following rapamycin exposure was evident when compared with a baseline U87MG cell, which featured very intense α-syn immuno-fluorescence. In Figure 5, the single α-syn and its co-staining with DAPI indicate a strong nuclear placement of α-syn immunostaining. This contrasts markedly with the negligible α-syn immuno-fluorescence observed in astrocytes (Figure 5). Rapamycin decreased α-syn immuno-fluorescence dose-dependently, as presented in the graph. In detail, while the dose of 1 nM was slightly effective, the dose of 10 nM was fully effective in suppressing α-syn immuno-fluorescence (Figure 5). This corresponds to a dose fully effective in inhibiting mTOR (Figure 4). Higher doses of rapamycin did not further contribute to the decrease in α-syn levels, which approached the amount of α-syn detected within astrocytes in baseline conditions (Figure 5).

### 3.5. Total and PK-Resistant Immunostaining for α-Syn Is Suppressed by Rapamycin

**Figure 6:**
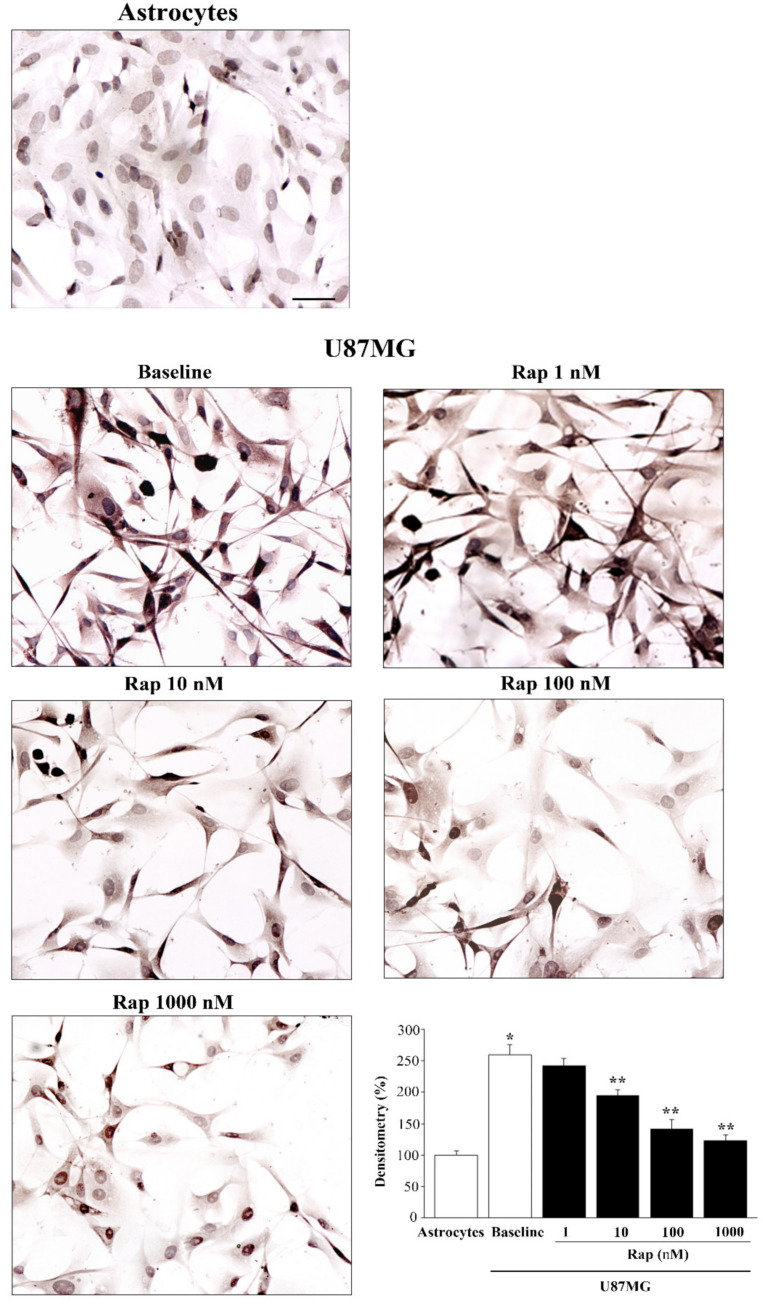
Rapamycin dose-dependently reduced α-syn-immunostaining. Representative pictures of α-syn immuno-positive astrocytes and U87MG cells in baseline conditions and following rapamycin (Rap) administration. The graph reports densitometry for α-syn immunostaining within astrocytes compared with U87MG cells, in baseline conditions and following rapamycin administration. Data are given as the mean percentage ± SEM of optical density from *n* = 30 cells obtained in *N* = 3 independent experiments (assuming the controls to be 100%). * *p* < 0.05 compared with astrocytes; ** *p* < 0.05 compared with baseline and Rap 1 nM. Scale bar = 22.5 μm.

Similar to α-syn when detected by immunofluorescence, α-syn detected by immuno-peroxidase decreased with increasing doses of rapamycin (Figure 6). Thus, the slight signal, which was detectable in astrocytes in baseline conditions, was comparable with U87MG cells treated with the highest dose of rapamycin (similar in Figure 5 and Figure 6). When comparing these immuno-peroxidase results with those obtained from immuno-fluorescence, there was a slightly progressive decrease in α-syn, even for doses higher than 10 nM, which could not be detected by fluorescence. This is likely to depend on the specific technique used during light microscopy. Only a stoichiometric quantitative assessment of the protein, as reported in Section 3.8, can firmly establish the details of such a dose-dependency.

With immuno-peroxidase, rapamycin at the dose of 10 nM decreased α-syn immunostaining to 74.9% ± 3.7% of baseline (Figure 6), while 100 nM rapamycin decreased α-syn to 54.6% ± 5.5% of baseline, and 1000 nM rapamycin decreased α-syn to 47.5% ± 3.5% of baseline, with the amount detected in baseline astrocytes being 38.5% ± 2.5% of that occurring in baseline U87MG cells (graph of Figure 6).

**Figure 7:**
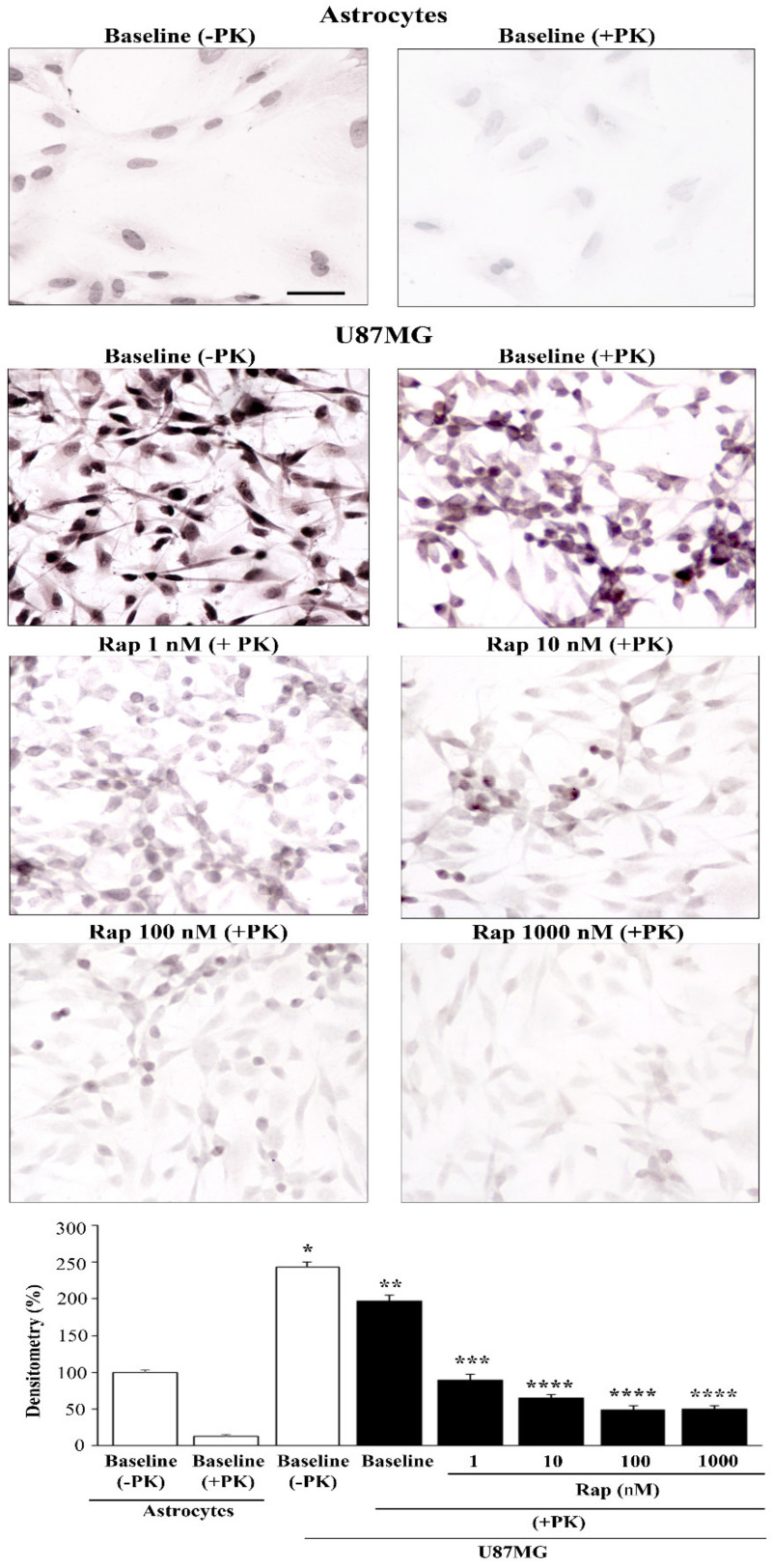
Rapamycin dose-dependently suppressed PK-resistant α-syn-immunostaining. Representative pictures of total (−PK) and PK-resistant (+PK) α-syn immunostaining within astrocytes and U87MG cells, in baseline conditions (baseline) and following increasing doses of rapamycin (Rap). The graph reports densitometry for α-syn immunostaining within astrocytes compared with U87MG cells, in baseline conditions and following various doses of rapamycin. Data are given as the mean percentage ± SEM of optical density from *n* = 30 cells per group obtained in *N* = 3 independent experiments (assuming controls as 100%). * *p* < 0.05 compared with astrocytes (baseline −PK); ** *p* < 0.05 compared with baseline (−PK); *** *p* < 0.05 compared with baseline (+PK); **** *p* < 0.05 compared with baseline (+PK) and Rap 1 nM. Scale bar = 25.5 μm.

In order to assess whether high levels of α-syn occurring in U87MG cells were resistant to digestion with PK, in dedicated experiments, cells were exposed to PK to detect baseline levels of PK-resistant α-syn within astrocytes and U87MG cells (Figure 7).

Unlike astrocytes, where α-syn mostly consisted of PK-sensitive protein, in U87MG cells, most (80%) α-syn was PK-resistant (representative pictures in baseline conditions of Figure 7 and graph of Figure 7). This led to an excess of PK-resistant α-syn in U87MG cells compared with astrocytes, surpassing the difference in the total amount of α-syn. In fact, the excess PK-resistant α-syn approached a hundredfold that seen in astrocytes when measured using ultrastructural stoichiometry. It is remarkable that the rapamycin-induced suppression of α-syn levels was more pronounced for the PK-resistant isoform (representative pictures and graph of Figure 7) compared with total α-syn (Figure 6). Again, in the case of PK-resistant α-syn, the dose of 1 nM already produced a significant effect, while a 10 nM dose of rapamycin was enough to reach the plateau. In fact, the percentage of PK-resistant α-syn immunostaining in controls was 81.1% ± 3.2% of baseline (−PK) U87MG cells, while the percentage of PK-resistant α-syn immunostaining in U87MG cells exposed to 1 nM, 10 nM, 100 nM, and 1000 nM rapamycin was 36.8% ± 3.3%, 26.6% ± 1.9%, 19.8% ± 2.2%, and 20.4% ± 1.8%, respectively. This suggests that PK-resistant α-syn is very sensitive to mTOR inhibition.

Representative pictures reported in Figure 6 and Figure 7 confirm the concomitant morphological changes induced by increasing doses of rapamycin in U87MG cells, such as the loss of the typical fusiform cell body toward a pyramidal cell body, increased cell branching, and nuclear volume.

### 3.6. Rapamycin Suppresses Both Total and PK-Resistant α-Syn with Immunoblotting

**Figure 8:**
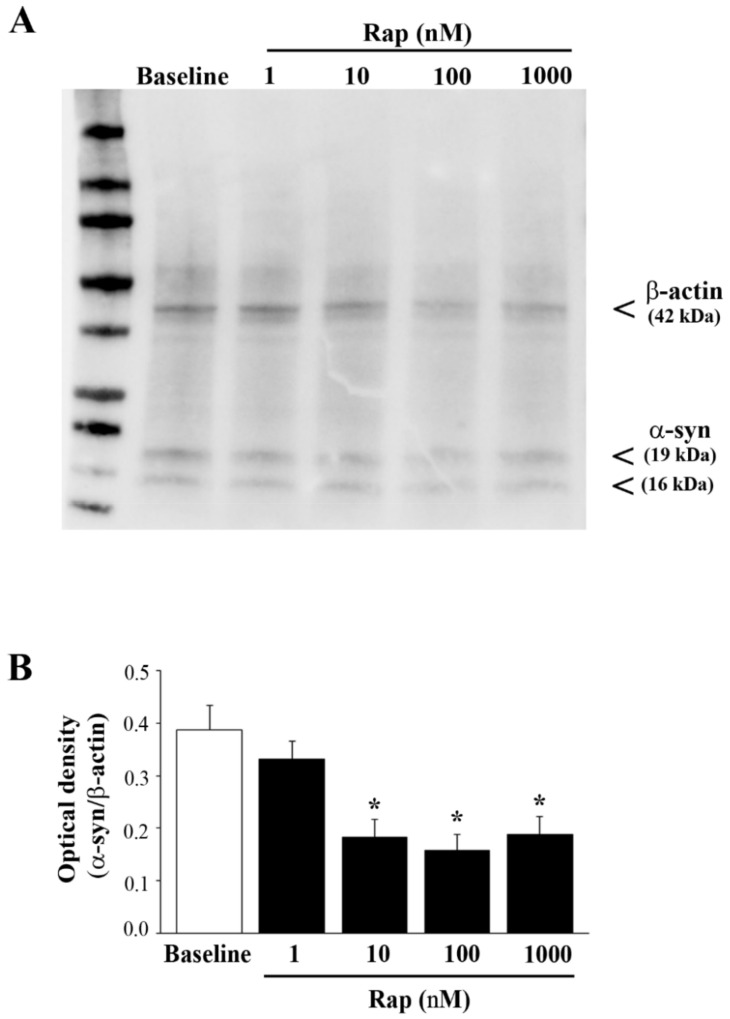
Rapamycin reduced α-syn assessed by immunoblotting. (**A**) Representative immunoblotting for α-syn and the housekeeping β-actin in baseline conditions (baseline) and in rapamycin-treated U87MG cells. (**B**) The ratio between the optical densities of α-syn and β-actin is reported in the graph. Data are given as the mean ± SEM of the optical density of *n* = 5 values for each experimental group, measured in *N* = 5 independent experiments. The original figure of Figure 8A can be found in Supplementary Figure S4. * *p* < 0.05 compared with baseline.

To confirm the occurrence of PK-resistant α-syn, control and rapamycin-treated cells were exposed to a PK-denaturing agent; then, immediately after completing electrophoretic separation, the protein bands were visualized using a stain-free technology on gels (Supplementary Figure S3). In order to assess the rapamycin-induced suppression of α-syn levels in U87MG cells, SDS-PAGE immunoblotting was carried out (Figure 8A), which confirmed the data reported in Figure 6. To be specific, semi-quantitative measurement based on the optical density of the ratio between immuno-positive bands of α-syn vs. β-actin in U87MG cells (Figure 8B) replicated the data reported in Figure 6. Remarkably, when using PK, the amount of α-syn that remained detectable possessed a different band pattern, which suggests that PK altered the aggregation of the protein in the sample, although this point remains to be established.

**Figure 9:**
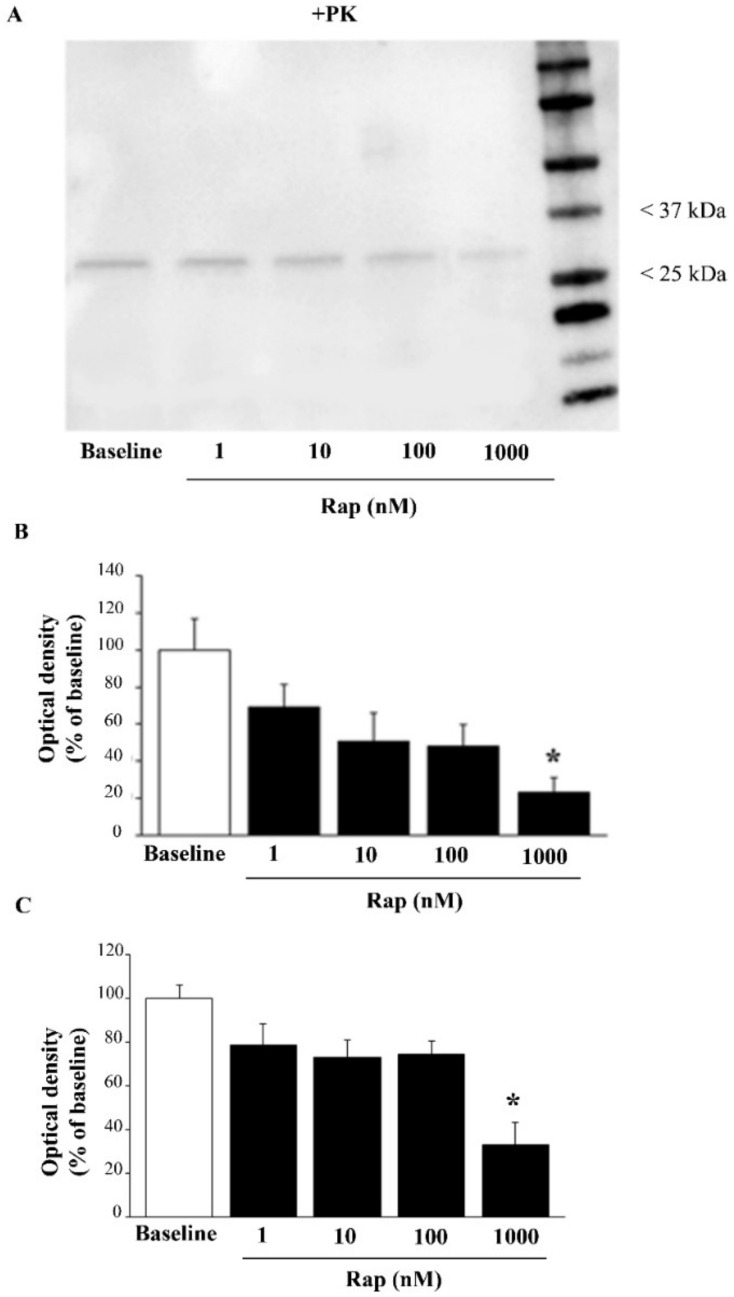
Rapamycin reduced the amount of PK-resistant α-syn. (**A**) Representative PK-resistant immunoblotting of α-syn in baseline conditions (baseline) and in rapamycin-treated U87MG cells (data were validated in *N* = 3 different gels). Graphs report densitometry as a percentage of baseline (assuming the baseline to be 100%) (**B**) or as a ratio between PK-resistant α-syn and β-actin (as in the gel without PK treatment), assuming the baseline to be 100% (**C**). Data are given as the mean percentage ± SEM of optical density of *n* = 3 blots for each experimental group, measured in *N* = 3 independent experiments. The original figure of Figure 8A can be found in Supplementary Figure S5. * *p* < 0.05 compared with baseline.

The effects of rapamycin on PK-resistant α-syn are shown as representative blots in Figure 9A, while the graph in Figure 9B indicates that the suppression of PK-resistant α-syn produced by rapamycin was more marked compared with total α-syn (compare the graphs of Figure 8B and Figure 9B,C), confirming the results of immuno-cytochemistry given in Figure 7. An alternative quantification of the PK-resistant α-syn is provided in Supplementary Figure S6. It is remarkable that, even with immunoblotting, there was already an effect at 1 nM rapamycin, which was more evident for the +PK α-syn, compared with the −PK α-syn. Again, the band pattern of α-syn immunoblots was modified by rapamycin, suggesting that autophagy induced an alteration in the protein band pattern, which may be derived from altered aggregation, as addressed in Figure 8.

### 3.7. Silencing α-Syn Expression Does Not Alter Cell Viability While Producing Phenotypic Changes

**Figure 10:**
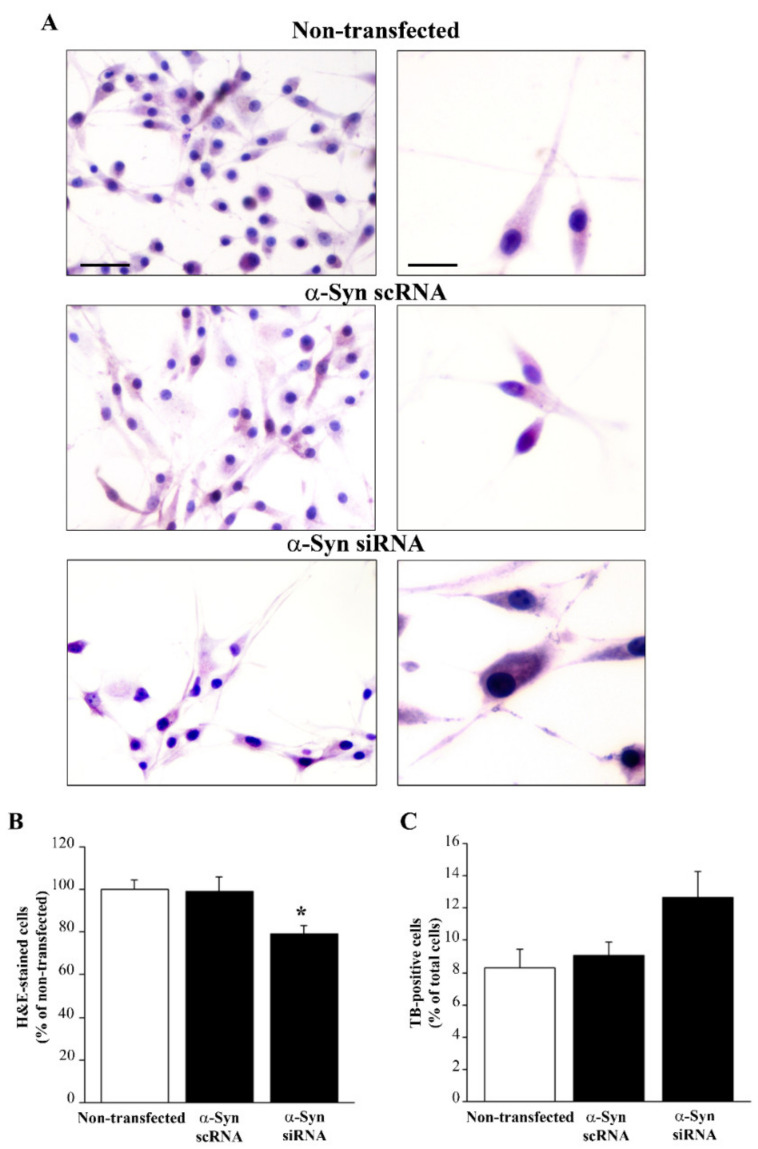
Silencing α-syn did not alter cell viability while producing phenotypic changes. (**A**) Representative pictures are shown of H&E-stained U87MG cells from non-transfected, α-syn scRNA-transfected, and α-syn siRNA-transfected cells. Graphs report the percentage of H&E-stained cells (considering non-transfected cells to be 100%) (**B**) and the percentage of TB-positive cells among total cells (**C**). Values are given as the mean percentage ± SEM of *n* = 6 counts obtained in *N* = 3 independent experiments. * *p* < 0.05, compared with non-transfected cells. Scale bars = 70 μm (low magnification); 30 μm (high magnification).

**Figure 11:**
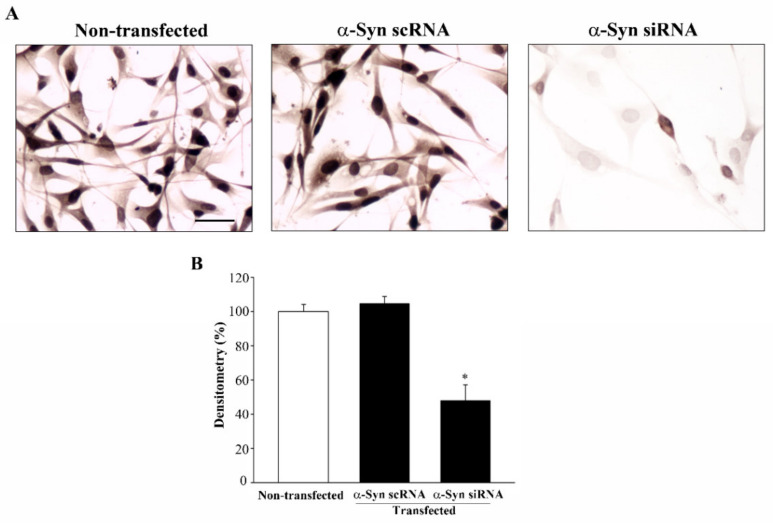
Silencing α-syn significantly reduced the nestin immunostaining. (**A**) Representative pictures are shown of nestin immuno-peroxidase from non-transfected, α-syn scRNA-transfected, and α-syn siRNA-transfected cells. (**B**) Graph reports the densitometry measured in the whole cells. Data are given as the mean percentage ± SEM of *n* = 30 counts obtained in *N* = 3 independent experiments. * *p* < 0.05 compared with non-transfected cells. Scale bar = 35 μm.

In order to investigate the effects of the lack of α-syn in GBM cells, U87MG cells were transfected with α-syn siRNA, which reduced α-syn expression (as shown by immunoblotting for α-syn in Supplementary Figure S7). Analysis under light microscopy of silenced U87MG cells after H&E staining shows that silencing α-syn expression induced slight phenotypic changes (Figure 10A) that markedly resembled the morphological changes induced by rapamycin (Figure 6 and Figure 7). In these silenced U87MG cells, the counts of H&E-stained cells revealed a decrease in the total number of U87MG cells compared with non-transfected and scRNA-transfected cells (Figure 10B). The TB assay revealed no reduction in cell viability in α-syn-silenced cells (Figure 10C). This was also confirmed by Hoechst staining, which documented that the lack of α-syn did not result in increased apoptosis (Supplementary Figure S8). Silencing α-syn expression was also accompanied by a decrease in nestin immunostaining (Figure 11).

### 3.8. Ultrastructural Stoichiometry Quantifies Rapamycin-Induced α-Syn Decrease and Compartmentalization

**Figure 12:**
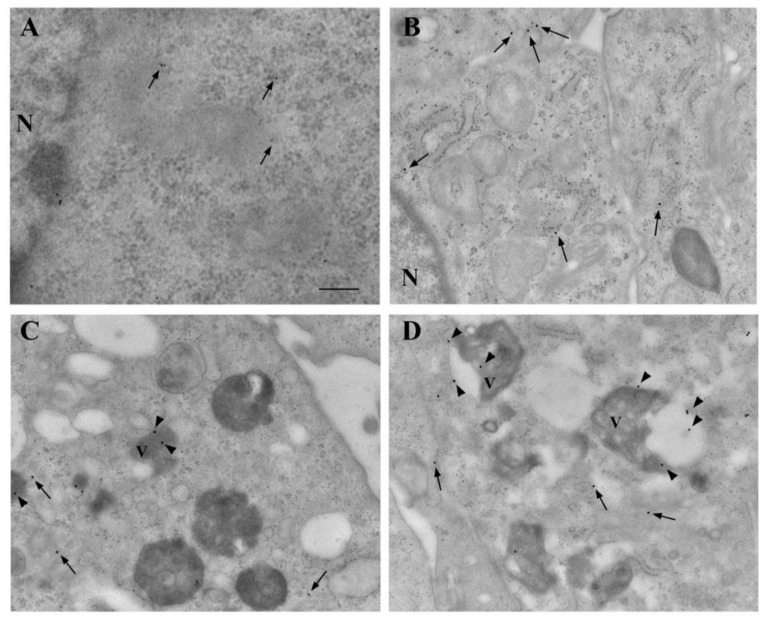
Rapamycin-induced α-syn vacuolar compartmentalization in U87MG cells. Representative micrographs showing astrocytes (**A**) and U87MG cells (**B**), in baseline conditions and following rapamycin exposure at 10 nM (**C**) and 100 nM (**D**). In astrocytes, α-syn was rarely found within the cytosol, nucleus, and vacuoles, whereas, in U87MG cells, α-syn immuno-gold was abundantly present in the cytosol and nucleus, with negligible vacuolar compartmentalization. Rapamycin reduced α-syn in the cell and increased its placement within the vacuoles. Arrows = α-syn immuno-gold particles in the cytosol; arrowheads = α-syn immuno-gold particles within the vacuoles. N = nucleus; V = vacuole. Scale bar = 0.28 μm.

This analysis was carried out by specifically using 10 nM and 100 nM rapamycin. In representative astrocytes (Figure 12A) and U87MG cells (Figure 12B), a decrease in α-syn immuno-gold was observed following rapamycin exposure. In baseline conditions, α-syn immuno-gold particles were abundantly dispersed in the cytosol of U87MG cells (Figure 12B). Following rapamycin exposure, the α-syn immuno-gold particles increased the compartmentalization within vacuoles (see the arrowheads in the representative micrographs of Figure 12C,D).

**Figure 13:**
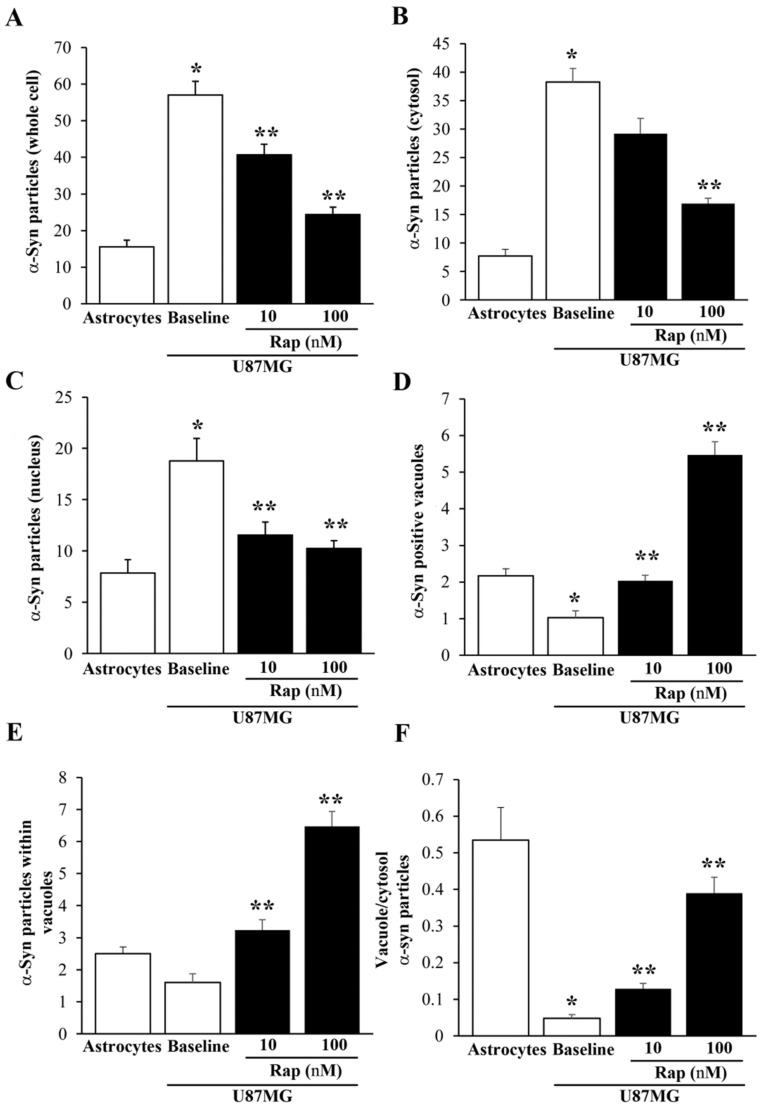
Counts of α-syn immuno-gold within the astrocytes and U87MG cells. Graphs report the number of α-syn immuno-gold particles within the whole cell (**A**), cytosol (**B**), and nucleus (**C**). The number of α-syn positive vacuoles is reported in (**D**), while the mean number of α-syn immuno-gold particles within each vacuole is reported in (**E**). The ratio of α-syn immuno-gold particles within vacuoles vs. the cytosol is reported in (**F**). These data were obtained from the astrocytes and U87MG cells, in baseline conditions and following rapamycin exposure (Rap 10 nM and Rap 100 nM). Data are given as the mean ± SEM from *n* = 30 cells per experimental group, obtained in *N* = 3 independent experiments. * *p* < 0.05 compared with astrocytes; ** *p* < 0.05 compared with baseline.

As shown in the graphs in Figure 13, the effects of rapamycin on α-syn levels and compartmentalization were assessed via immuno-gold counts. In detail, the graph of Figure 13A reports that α-syn was much more abundant within U87MG cells than astrocytes, and that rapamycin reduced the amount of α-syn more in the U87MG cells in the cytosol (Figure 13B) than in the nucleus (Figure 13C). Within the cytosol, rapamycin increased the α-syn immuno-positive vacuoles (see the graph in Figure 13D) and the number of α-syn immuno-gold particles within each vacuole (see the graph in Figure 13E). In this way, despite rapamycin decreasing the amount of total α-syn in U87MG cells, it allocated α-syn within autophagy-like vacuoles, as shown by the ratio of α-syn within vacuoles vs. α-syn within the cytosol, which was increased by rapamycin (Figure 13F). These data indicate that rapamycin decreased the α-syn within U87MG cells but increased the α-syn compartmentalization within the vacuoles, which may represent the organelles responsible for the autophagy-induced clearance of α-syn.

**Figure 14:**
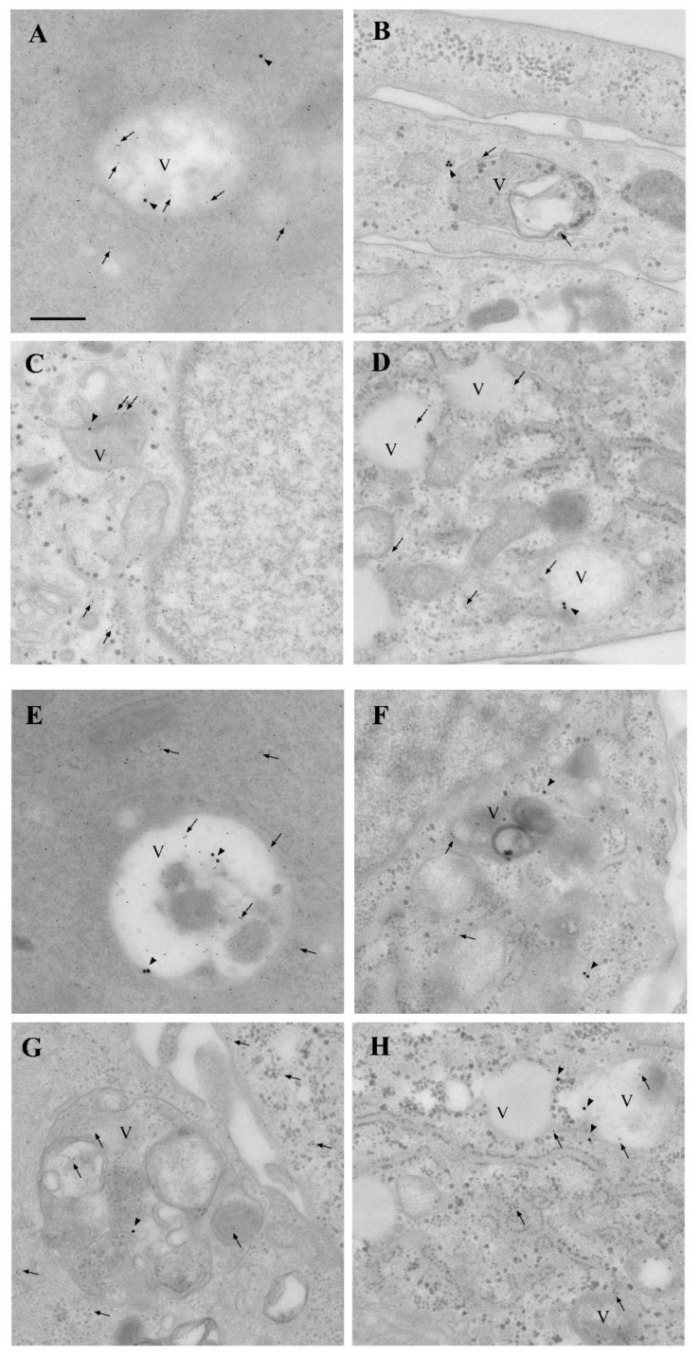
Rapamycin-induced α-syn + LC3- and α-syn + cathepsin D-positive vacuoles in GBM cells. Representative micrographs showing astrocytes (**A**,**E**) and U87MG cells (**B**,**F**), in baseline conditions and following rapamycin exposure at 10 nM (**C**,**G**) and 100 nM (**D**,**H**). Immuno-gold for α-syn (20 nm) and LC3 (10 nm) is shown in (**A**–**D**), while immuno-gold for α-syn (20 nm) and cathepsin D (10 nm) is shown in (**E**–**H**). Arrowheads = α-syn immuno-gold particles; arrows = LC3 or cathepsin D immuno-gold particles. V = vacuole. Scale bar = 0.15 μm.

**Figure 15:**
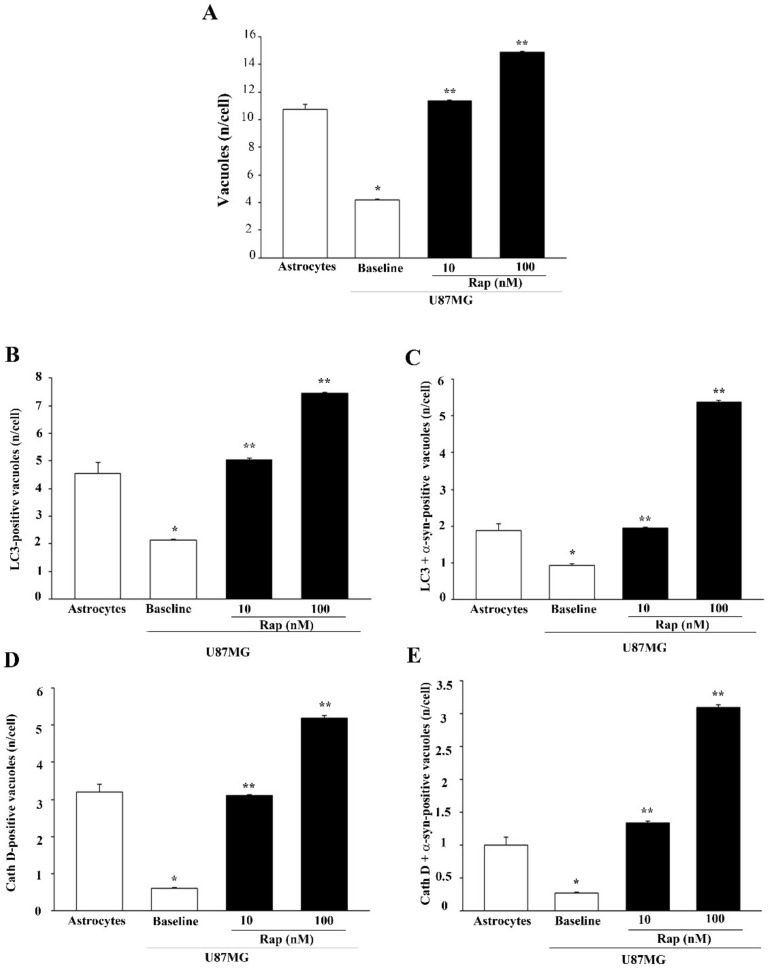
Quantification of α-syn + LC3- or α-syn + cathepsin D-positive vacuoles within astrocytes and U87MG cells in baseline conditions and after rapamycin. Graphs report the total number of vacuoles per cell (**A**), the number of LC3-positive vacuoles per cell (**B**), the number of LC3 + α-syn-positive vacuoles per cell (**C**), the number of cathepsin D-positive vacuoles per cell (**D**), and the number of cathepsin D + α-syn-positive vacuoles per cell (**E**). These data were obtained from astrocytes and U87MG cells in baseline conditions and following rapamycin exposure (Rap 10 nM and Rap 100 nM). Data are given as the mean ± SEM from *n* = 30 cells per experimental group obtained in *N* = 3 independent experiments. * *p* < 0.05 compared with astrocytes; ** *p* < 0.05 compared with baseline.

In order to better identify α-syn-positive vacuoles, immuno-electron microscopy for α-syn, analyses of the autophagy marker LC3, and the lysosomal marker cathepsin D (Cath D) were carried out in astrocytes and U87MG in baseline conditions and following rapamycin exposure (Figure 14). In U87MG cells, rapamycin treatment (10 nM and 100 nM) increased the colocalization of α-syn immuno-gold particles, both with LC3 (Figure 14C,D) and cathepsin D (Figure 14G,H) particles, compared with the baseline (Figure 14B). This colocalization occurred within the vacuoles and was quantified through stoichiometry, as shown in the graphs in Figure 15. These measurements show that rapamycin, while increasing the number of total vacuoles, specifically augmented the vacuolar colocalization of α-syn with both LC3 and cathepsin D (Figure 15).

### 3.9. Ultrastructural Stoichiometry Quantifies Rapamycin-Dependent Decrease in PK-Resistant α-Syn and α-Syn Compartmentalization

**Figure 16:**
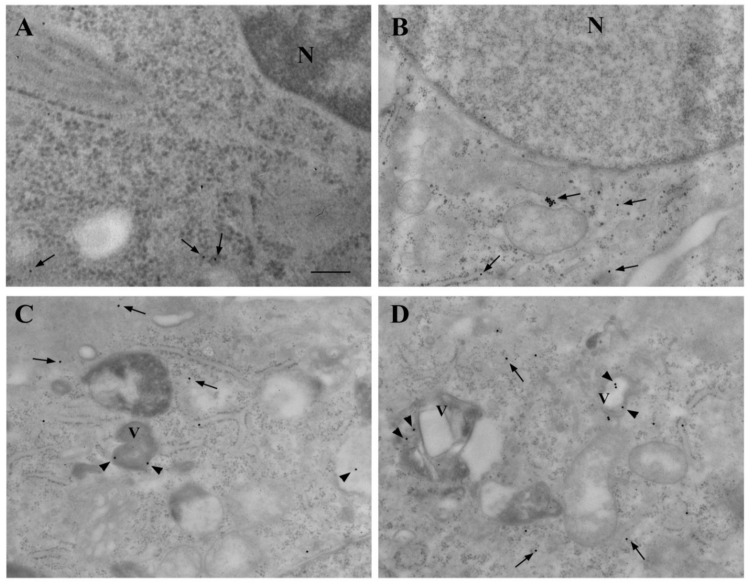
Rapamycin-induced vacuolar compartmentalization of PK-resistant α-syn in U87MG cells. Representative micrographs show the presence of PK-resistant isoform of α-syn within astrocytes (**A**) and U87MG (**B**–**D**) cells, in baseline conditions (**A**,**B**) and following rapamycin exposure (Rap 10 nM and Rap 100 nM, (**C**,**D**), respectively). Arrows = α-syn immuno-gold particles in the cytosol; arrowheads = α-syn immuno-gold particles within vacuoles. N = nucleus; V = vacuole. Scale bar = 0.28 μm.

As shown in representative micrographs, following PK pretreatment, the amount of α-syn was barely detectable within astrocytes (Figure 16A), while it was abundant within U87MG cells in baseline conditions (Figure 16B). Rapamycin suppressed PK-resistant α-syn (Figure 16C,D) while increasing the compartmentalization of PK-resistant α-syn within vacuoles.

**Figure 17:**
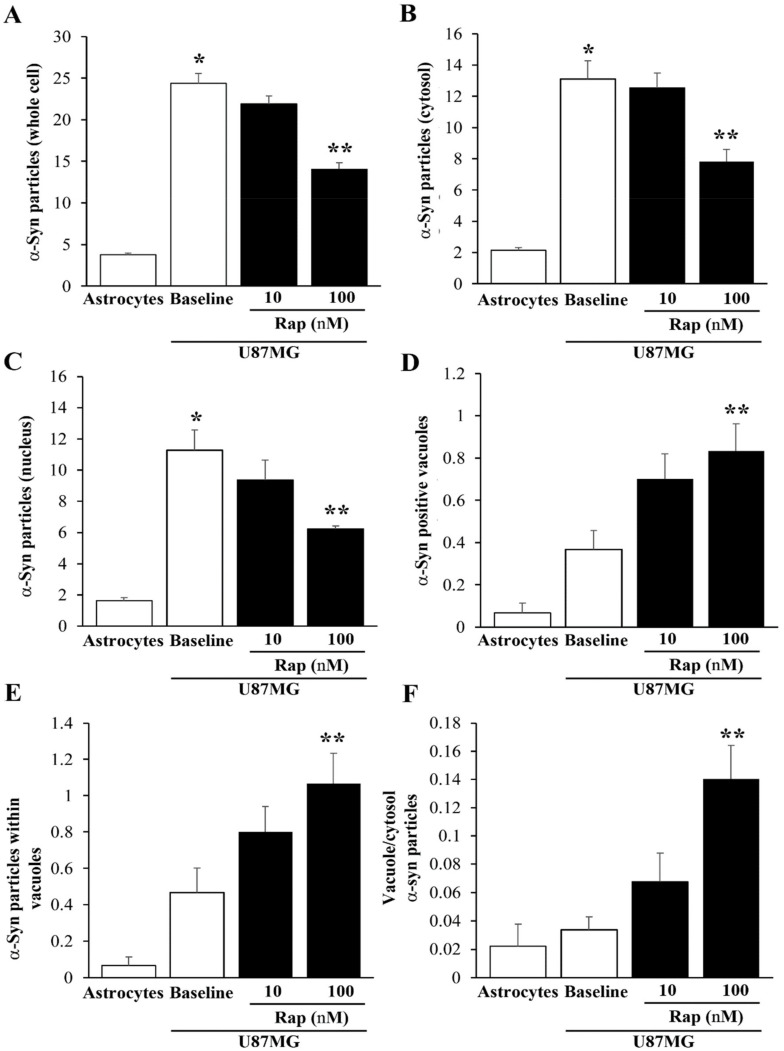
Count of PK-resistant α-syn immuno-gold within astrocytes and U87MG cells. Graphs report the number of α-syn immuno-gold particles within the whole cell (**A**), cytosol (**B**), and nucleus (**C**). The number of α-syn-positive vacuoles is reported in (**D**), while the mean number of α-syn immuno-gold particles within each vacuole is reported in (**E**). The ratio of α-syn immuno-gold particles within vacuoles vs. the cytosol is reported in (**F**). These data were obtained from astrocytes and U87MG after PK treatment in baseline conditions and following rapamycin exposure (Rap 10 nM and Rap 100 nM). Data are given as the mean ± SEM from *n* = 30 cells per experimental group, obtained from *N* = 3 independent experiments. * *p* < 0.05 compared with astrocytes; ** *p* < 0.05 compared with baseline.

As shown in the graphs of Figure 17, these effects were quantified by counting PK-resistant α-syn in baseline conditions in the whole cell (Figure 17A), the cytosol (Figure 17B), and the nucleus (Figure 17C). The amount of PK-resistant α-syn counted in U87MG cells was roughly 80% of that counted without PK, indicating how such an isoform was predominant in U87MG cells, as documented through different methods. Rapamycin increased the number of α-syn-positive vacuoles (Figure 17D), as well as the mean number of α-syn immuno-gold per vacuole (Figure 17E), while it increased the ratio of vacuole/cytosol α-syn dose-dependently (Figure 17F).

### 3.10. α-Syn Gene Expression Is Reduced by Rapamycin in U87MG Cells

**Figure 18:**
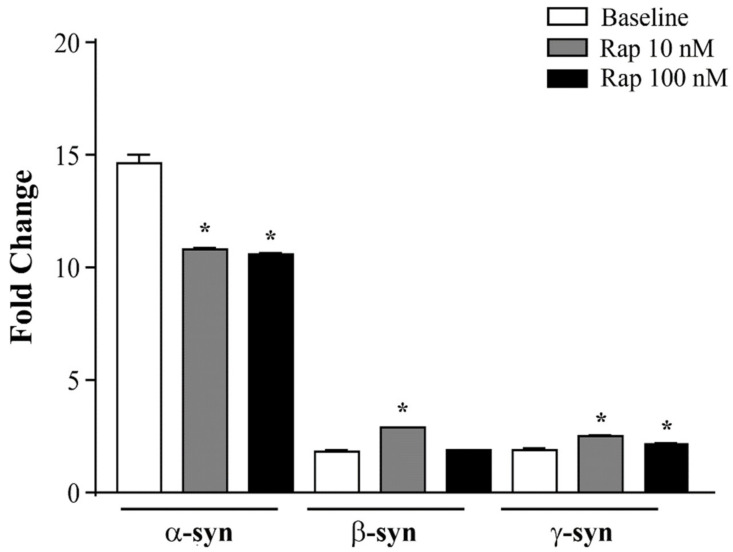
Effects of rapamycin on the mRNA levels of α-syn, β-syn, and γ-syn in U87MG cells. α-Syn, β-syn, and γ-syn gene transcripts were assessed by quantitative RT-PCR analysis in U87MG cells in baseline conditions (baseline), and following rapamycin exposure (Rap 10 nM and Rap 100 nM). Data are given as the mean ± SEM from *n* = 4 samples from *N* = 2 independent experiments, normalized with two different internal references, globin, and actin. * *p* < 0.05 compared with baseline.

The ΔΔCt analysis of qRT-PCR indicates that rapamycin, at the doses of 10 and 100 nM, specifically decreased α-syn transcript levels, while β-syn and γ-syn mRNAs were not modified or increased (graph of Figure 18). This effect replicated that induced by rapamycin on α-syn native protein. This suggests that, in addition to improving α-syn clearance, rapamycin suppressed α-syn synthesis.

## 4. Discussion

The present study shows that, in baseline conditions, GBM cells featured a high amount of α-syn, which was substantially in excess compared with the α-syn levels measured within astrocytes in baseline conditions. These data were confirmed using various techniques. In fact, α-syn detection was carried out using immuno-peroxidase and immuno-fluorescence under light microscopy, as well as immunoblotting and stoichiometry under electron microscopy. The validation of primary antibodies against α-syn was thoroughly reported in Section 2.16. Nonetheless, in order to extend this evidence, RT-PCR was used to confirm the specificity of α-syn detection. However, caution is always needed, considering that primary commercial antibodies, despite being thoroughly investigated in the pilot validations (see Section 2 and Table 1), may recognize various synuclein isoforms.

In fact, previous investigations encompassing various brain tumors documented high levels of β-syn [46]. However, this was solely based on a single antibody procedure. The present study provides convergent evidence indicating α-syn overexpression in GBM cells. In fact, the remarkable difference between astrocytes and GBM cells in the synuclein family concerned only α-syn, although a slight but significant difference remained for β-syn and γ-syn, which were present in negligible amounts compared with α-syn. The high levels of α-syn occurred within whole GBM cells, compared with whole astrocytes. The faint staining detected in astrocytes is consistent with previous reports, indicating very low levels of α-syn within these cells [47,48,49,50]. The increase in α-syn within astrocytes has so far been described in the context of a fast-progressing, fast-spreading degenerative disorder known as multiple system atrophy, which tends to invade a number of brain areas [3,4,5,6,7]. When the amount of α-syn in the nucleus and cytosol of GBM cells was considered, both light and electron microscopy indicated that, within the cytosol of GBM cells, the amount of α-syn was more pronounced compared with the nucleus. This was consistently measured using different techniques. Quantitative stoichiometry roughly indicated a fivefold difference between the cytosol and the nucleus. The nuclear localization of α-syn, as originally described by Maroteaux et al. (1988) [51], has been confirmed by recent papers [52,53,54,55,56,57,58,59,60,61]. The significance of such an increase is likely to be relevant for neoplasms. In fact, recent studies have demonstrated that, in baseline conditions, α-syn has a role in DNA repair and its removal from the nucleus reduces the ability to repair DNA double-strand breaks that are induced by several insults [61]. The effective role of nuclear α-syn remains under debate. Some studies indicated that nuclear α-syn directly binds DNA and regulates gene expression [57,59,60]. Remarkably, nuclear α-syn includes various isoforms, encompassing phosphorylated and aggregated α-syn molecules, which vary in different (normal or pathological) conditions. The pattern of α-syn isoforms was replicated in this study, whereby α-syn bands at immunoblotting differed between GBM cells and astrocytes. This was clearly evident when the PK-resistant isoform was analyzed, although the significance of such a difference remains to be investigated concerning the amount of aggregation (as addressed in Section 5).

The amount of α-syn detected in GBM cells, which was in excess compared with astrocytes, was amplified when the PK-resistant isoform of α-syn was selectively measured. This was consistent throughout immune-histochemistry, immunoblotting, and ultrastructural stoichiometry since all procedures documented a GBM-dependent shift of α-syn toward a PK-resistant isoform. In fact, the amount of cytosol PK-resistant α-syn was substantially more in excess compared with total α-syn, when comparing GBM cells with astrocytes. In fact, the amount of PK-resistant α-syn represented 80% of total α-syn measured in GBM cells, while it was negligible in astrocytes. Again, in GBM cells, a shift occurred concerning the subcellular placement of α-syn. The occurrence of α-syn in baseline conditions within astrocytes, despite being scarce, was highly compartmentalized within autophagy-like and LC3-positive autophagy vacuoles, as well as cathepsin D-positive lysosomes, which contrasted the widespread cytosol distribution measured within GBM cells. Such a compartmentalization in baseline astrocytes was no longer present when ultrastructural stoichiometry was applied for counting PK-resistant α-syn. In fact, this nondigested isoform of the protein was homogeneously suppressed in astrocytes, independently of which cell compartment was considered.

Due to the high expression of mTOR that characterizes GBM cells, contributing to disease-spreading and malignancy, rapamycin was administered to assess to what extent such a high amount of α-syn could be mitigated by inhibiting mTOR. Doses of rapamycin of 10 nM and 100 nM were used on the basis of their efficacy in inhibiting mTOR activity (as measured by the mTOR downstream product, PS6). Both doses significantly mitigated the occurrence of α-syn within GBM cells. Depending on the method applied, some discrepancies were present in the dose–response curve. Nonetheless, all the procedures indicated that a dose of 10 nM produced a significant effect, which could reach the maximum (same levels as control astrocytes) according to immunofluorescence and RT-PCR. Thus, rapamycin at therapeutic doses reduced the amount of α-syn to values that were similar to those within astrocytes in baseline conditions. Significantly, the PK-resistant isoform of α-syn was suppressed more effectively by rapamycin compared with total α-syn. In detail, when the PK-resistant isoform was analyzed, the dose of 1 nM rapamycin had already presented significant effects. This indicates how crucial the mTOR pathway is for the fate of α-syn. In fact, the amount of α-syn, mostly its nondigested isoform, strongly depends on impaired autophagy activity due to mTOR overactivation. This mTOR-dependent autophagy suppression is typical within GBM cells, and, when it is reversed, sudden beneficial effects are induced [23,24,62]. In fact, it is remarkable that, concomitant to a decrease in α-syn levels, the phenotype of the GBM cell is modified by rapamycin to mimic an astrocyte-like shape. In fact, rapamycin concomitantly reverts the morphological changes of U87MG cells by promoting an increase in the size of the cell body, along with increased cell branching and nuclear volume. In addition, rapamycin suppresses nestin in GBM cells [23]. Similarly, phenotype reversal and a decrease in the stem-cell marker, nestin, were all induced by silencing α-syn. Again, the change in GBM phenotype induced by rapamycin was remarkable when also concerning α-syn compartmentalization. In fact, in addition to reducing the total amount of α-syn within GBM cells, α-syn protein particles were compartmentalized within autophagy-related vacuoles. These include both proper LC3-positive autophagy vacuoles and cathepsin D-positive lysosome vacuoles. Such a placement of α-syn within the autophagy/lysosome-related structures produced by rapamycin is to be expected, due to autophagy activation. Thus, the presence of a higher number of α-syn molecules within autophagy vacuoles under the effects of rapamycin is not surprising, even considering the net decrease in total α-syn levels within GBM cells. This is not a paradoxical finding and should rather be interpreted as a mechanistic effect, witnessed as part of an ongoing removal of α-syn through its shuttling toward autophagy–lysosomal clearance. This is most crucial when considering the remarkable amount of nondigested PK-resistant α-syn occurring in GBM cells. The amount and compartmentalization of α-syn measured in the present study in baseline GBM cells, compared with astrocytes, suggests a potential effect of this protein on the biology of GBM. The ability of rapamycin to decrease α-syn is likely to extend beyond an accelerated protein clearance. In fact, the fascinating evidence that even the primary transcript of α-syn was decreased by rapamycin administration opens novel pathways through which mTOR suppression may act at an epigenetic level. The epigenetics of rapamycin is likely to involve a number of loci and may be key in remodeling cell biology [63,64]. These data help to contextualize novel evidence to foster dedicated investigations. In turn, α-syn possesses a strong effect of DNA methylation, as recently demonstrated, which determines the powerful stimulation of cell proliferation [57,65,66,67,68,69,70,71].

In line with this finding, mutations in α-syn alter the cell proliferation within the subependymal ventricular zone [72], which is likely to depend on the epigenetic effects promoted by α-syn [65].

It is likely that, in those cells where mTOR is upregulated and autophagy is suppressed, the eventual accumulation of α-syn triggers a biochemical cascade that affects gene expression [65,73], cell proliferation [71], and cell differentiation [74]. This shifts the original question of investigating whether α-syn is highly expressed in GBM to the following question: does α-syn take an active role in the neurobiology of GBM?

In line with this finding, a recent study disclosed the pivotal role of α-syn in the genesis of melanoma, a specific tumor of the skin, which originates from melanocytes, a cell type that, analogously to astrocytes, is derived from neural crests. Melanoma cells feature the high expression of α-syn [74,75,76,77,78]. The involvement of α-syn in some tumors derived from cells originating from neural crests leads to a hypothesis that an increase in α-syn may work as a trigger of both degeneration and abnormal proliferation, depending on the cell isotype in which it occurs.

In this scenario, it becomes crucial to establish which cell mechanism may induce a primary increase in α-syn. Since the amount of α-syn critically depends on the status of protein-clearing pathways and mostly on autophagy [14], emphasis should be placed on those conditions featuring autophagy suppression. This is the case for disorders featuring mTOR upregulation, since such a molecular complex is a strong autophagy suppressor. In GBM, the impressive overactivation of mTOR, which suppresses autophagy [15,16,17,18], plays a mechanistic role in tumor progression. To be specific, impairment of the autophagy machinery is key in fostering tumor initiation and enlargement. In fact, the amount of autophagy suppression is directly related to the malignancy of GBM [19,20,21,22]. Due to the strong role of autophagy in removing α-syn and the unique suppression of autophagy in GBM, it is surprising that no study has so far investigated the occurrence of α-syn in GBM cells. The present study indicated that, despite a small increase in β-syn in GBM cells compared with astrocytes, such a difference was substantial with respect to α-syn. RT-PCR demonstrated that, under the influence of rapamycin, which produced a regression in the tumor, α-syn was suppressed, while β-syn and γ-syn were slightly increased. Again, this suggests a role specific to α-syn, compared with β-syn and γ-syn, in association with tumor malignancy. This is consistent with the role of α-syn in the physiology of the cell since α-syn is strongly implicated in promoting cell growth [55,66,68,71,76] and the cell cycle [70]. Thus, it is not surprising that α-syn may be abundantly expressed in cancers [75,79,80] including brain tumors [46,81]. One should further consider that the overexpression of α-syn, in turn, inhibits autophagy through mTOR activation, which may lead to a vicious cycle [82]. Conversely, rapamycin mitigates mTOR overactivation, thereby promoting autophagy, which was also occluded due to overexpressed α-syn [82,83].

The specific role of α-syn within human GBM remains to be investigated in a number of GBM cell types since the protein may represent a specific marker of certain cell phenotypes within the tumor. Conflicting data have been reported concerning α-syn gene expression and α-syn protein expression, which may reflect tumor and antibody specificity [46]. At any rate, the recovery of a differentiated phenotype and the suppression of the stem-cell marker nestin that occurs in GBM cells, following the silencing of α-syn, implicate an active role of the protein in the biology of GBM.

## 5. Conclusions

The present manuscript indicates that, in baseline conditions, GBM cells feature high levels of α-syn compared with astrocytes. This was confirmed using various antibody-based procedures, including immuno-peroxidase and immuno-fluorescence under light microscopy, Western blotting, and immuno-gold transmission electron microscopy. This was further validated by measuring a higher amount of the primary transcript for α-syn in GBM cells compared with astrocytes. This was substantially in excess compared with the slight differences measured for β-syn and γ-syn. In keeping with present data, the amount of α-syn was significantly suppressed by rapamycin-induced mTOR inhibition in GBM cells. This also occurred for the primary transcript of α-syn, while the transcript levels of β-syn and γ-syn were not affected or increased.

The greater amount of α-syn detected in GBM cells compared with astrocytes was magnified when the PK-resistant isoform of α-syn was measured. In fact, the amount of cytosol PK-resistant α-syn further exceeded that level measured within astrocytes. This may suggest that α-syn is present as a protein aggregate within GBM cells, as reported in recent findings. These studies showed that the α-syn molecules that are still detectable after PK digestion correspond to aggregated α-syn [84]. This latter study, using 2D and 3D analyses of α-syn in cell cultures, showed that cytosol α-syn aggregates are mostly proteinase K (PK)-resistant. In line with this finding, Arsac et al. [85] demonstrated that, in the absence of aggregated α-syn molecules, phosphorylated or misfolded α-syn does not resist PK treatment. Similarly, PK digestion allowed the detection of aggregated α-syn, according to the study by Takaichi et al. [86]. These authors emphasized that α-syn aggregates can only be detected following PK digestion. Therefore, the PK-resistant core of α-syn-containing structures was isolated to enable the analysis of the structure of α-syn aggregates using mass spectrometry [87].

Nonetheless, here, we wish to be cautious in establishing an equivalence between PK-resistant immuno-reactivity for α-syn and α-syn aggregates. In fact, it cannot be assumed that total PK-resistant α-syn corresponds to α-syn aggregates. Thus, further analysis using mass spectrometry is needed to establish the amount and structure of α-syn aggregates that correspond to the fraction of PK-resistant α-syn.

Therefore, we suggest giving these findings the benefit of the doubt when considering the significance of PK-resistant α-syn in the relationship between α-syn aggregates.

The results of the present study provide a novel marker for GBM cells beyond classic cancer stem-cell markers and confirm a role of α-syn in CNS pathology that goes beyond neurodegeneration to embrace the dysregulation of cell growth as it occurs in GBM. In keeping with this finding, it will be important to investigate whether, by occluding the expression of α-syn, the classic stem-cell markers that typically stain GBM cells are concomitantly suppressed. This evidence is presently being investigated. In line with a potentially active role of α-syn in the course of GBM, it is worth assessing whether α-syn may also spread from GBM cells to astrocytes, to prime their expression of stem-cell markers.

## Figures and Tables

**Table 1:** Technical comparison among the anti-α-syn primary antibodies used in this study.

Primary Antibody	Distributor	Cat#	RRID	Work Dilution
Rabbit polyclonal	Sigma	SAB4502828	AB_10746104	1:100 (IF) ^1^
Mouse monoclonal	Abcam	ab27766	AB_727020	1:1000 (IP)
Mouse monoclonal	BD Transduction Laboratories	610786	AB_398107	1:800 (WB)
Rabbit polyclonal	Sigma	SAB4502828	AB_10746104	1:50 (TEM)

^1^ IF = immuno-fluorescence; IP = immuno-peroxidase; WB = Western blotting; TEM = transmission electron microscopy.

## Data Availability

The data that support the findings of this study are available from the corresponding author upon reasonable request.

## Supplementary Materials

The following supporting information can be downloaded at: https://www.mdpi.com/article/10.3390/cancers14061382/s1, Figure S1: Negative control of the α-syn immunocytochemistry procedure, Figure S2: Immuno-blotting for α-syn in astrocytes and U87MG cells in baseline condition, Figure S3: Stain free gel following PK, Figure S4: Original gel for α-syn and the housekeeping protein α-actin shown in Figure 8, Figure S5: Original gel for α-syn and the housekeeping protein α-actin shown in Figure 9, Figure S6: Alternative quantification of immuno-blotting for PK-resistant α-syn with respect to the monomeric α-syn from PK-untreated samples, Figure S7: Immuno-blotting for α-syn in U87MG cells transfected with α-syn siRNA. Figure S8: Silencing α-syn in U87MG cells does not induce apoptosis.
