# Supplementary material for: Occurrence of Total and Proteinase K-Resistant Alpha-Synuclein in Glioblastoma Cells Depends on mTOR Activity
Source: Cancers (Basel). 2022 Mar 8;14(6):1382. doi: 10.3390/cancers14061382 (PMC8946689; doi:10.3390/cancers14061382)
Supplement: Supplementary file 1 [file cancers-14-01382-s001.zip › cancers-1573405-supplementary.pdf]

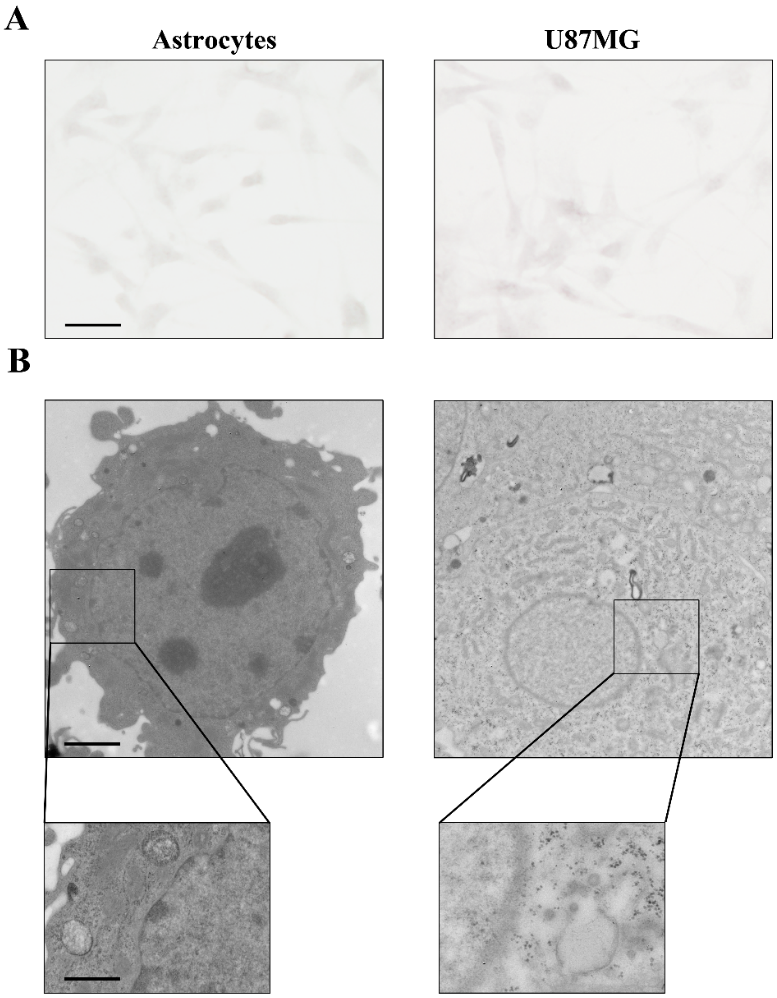

Figure S1. Negative control of the ⊚-syn immunocytochemistry procedure. Representative pictures from astrocytes and U87MG cells at light (**A**) and transmission electron microscopy (**B**) were obtained by omitting the anti-⊚-syn primary antibody. All pictures show the absence of immunoreaction. (**A**) Scale bar=35 m, (**B**) Scale bar = 0.6 ⊚m (low magnification); Scale bar = 0.15 m.

A

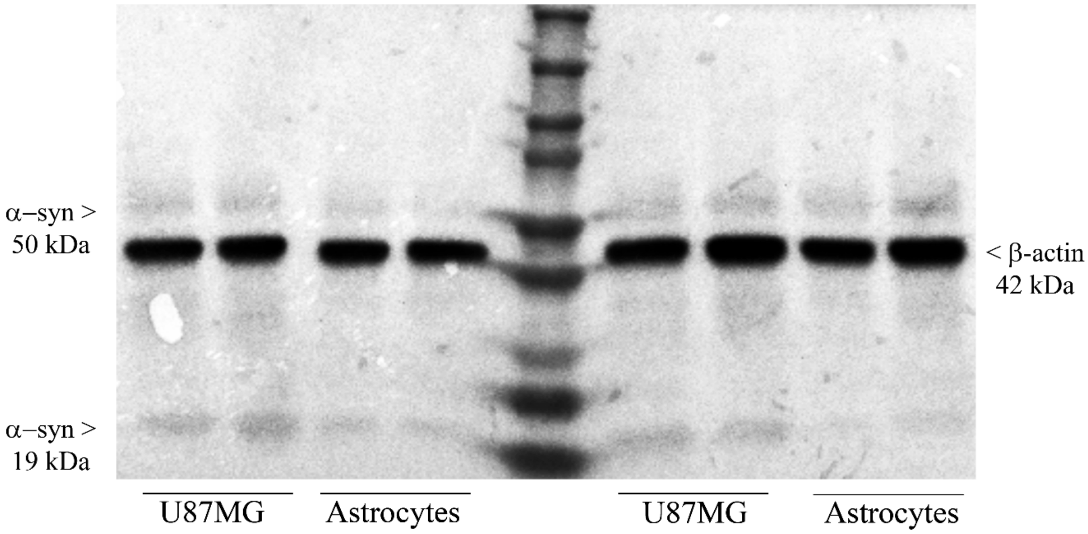

B

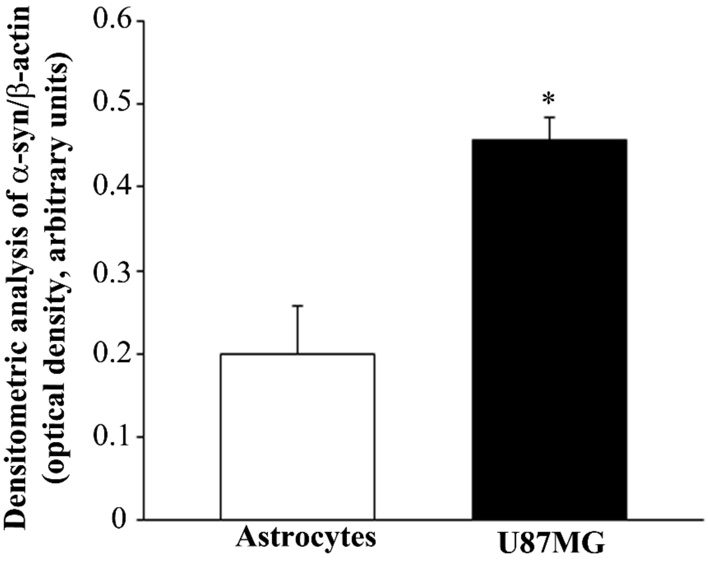

Figure S2. Immuno-blotting for a-syn in astrocytes and U87MG cells in baseline condition. (**A**) Representative immuno-blotting for ◎-syn and the housekeeping ◎-actin is shown. (**B**) The graph reports the related optical density. Values are given as the mean±S.E.M. of n = 3 blots obtained in N=1 experiment. * $p < 0.05$ compared with astrocytes. .

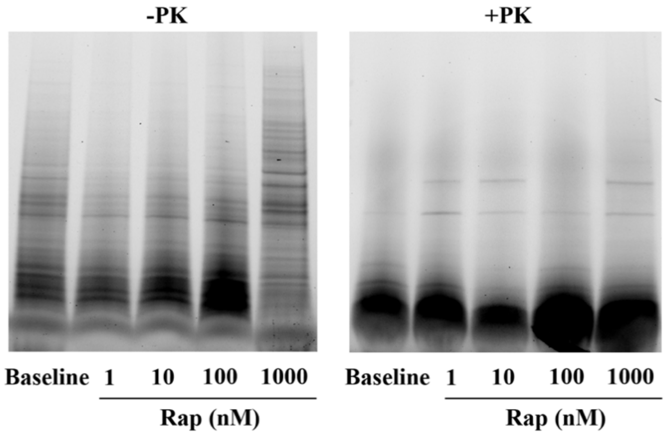

Figure S3. Stain free gel following PK. Representative images showing the pattern of protein bands visualized by using a stain-free gel enabled Bio-Rad UV transilluminator (ChemiDoc) without exposure to the denaturing agent (-PK) and following exposure to PK (+PK).

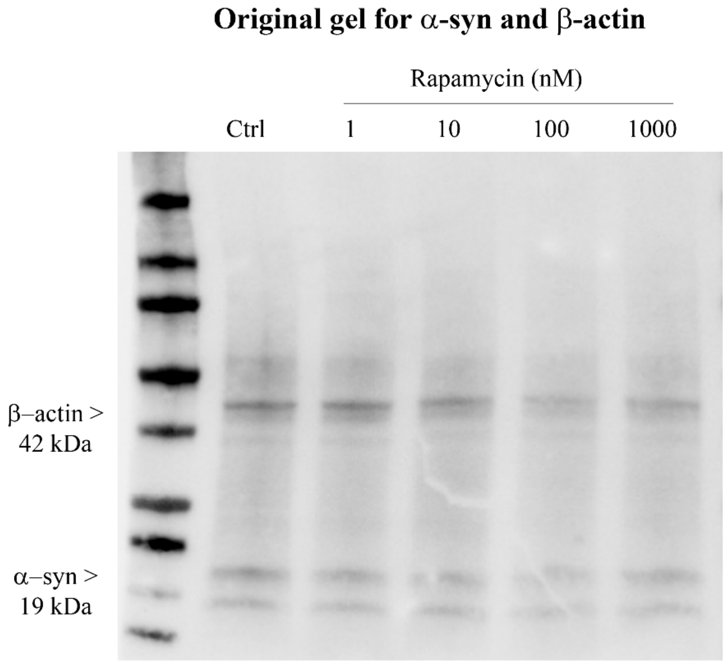

Figure S4. Original gel for ⊚-syn and the housekeeping protein ⊚-actin shown in Figure 8.

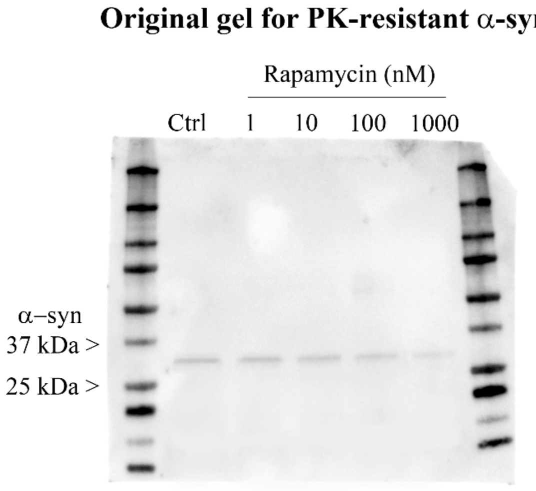

Figure S5. Original gel for ◎-syn and the housekeeping protein ◎-actin shown in Figure 9.

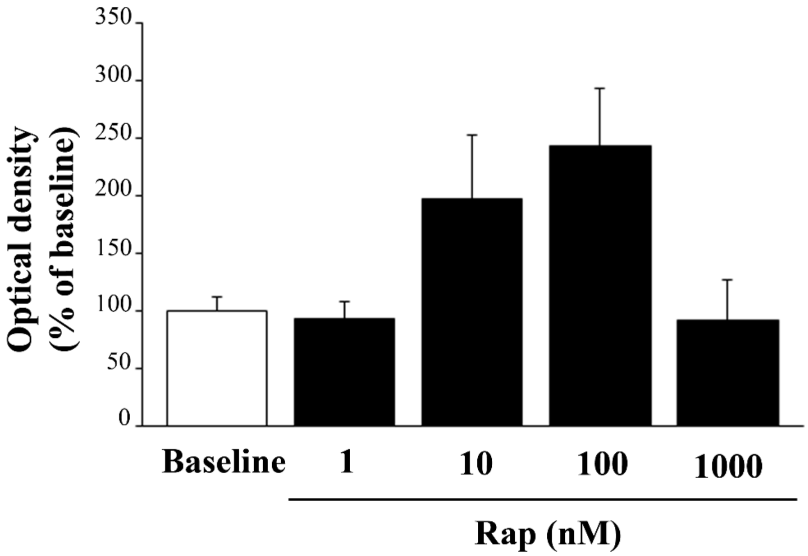

Figure S6. Alternative quantification of immuno-blotting for PK-resistant ◎-syn with respect to the monomeric ◎-syn from PK-untreated samples.

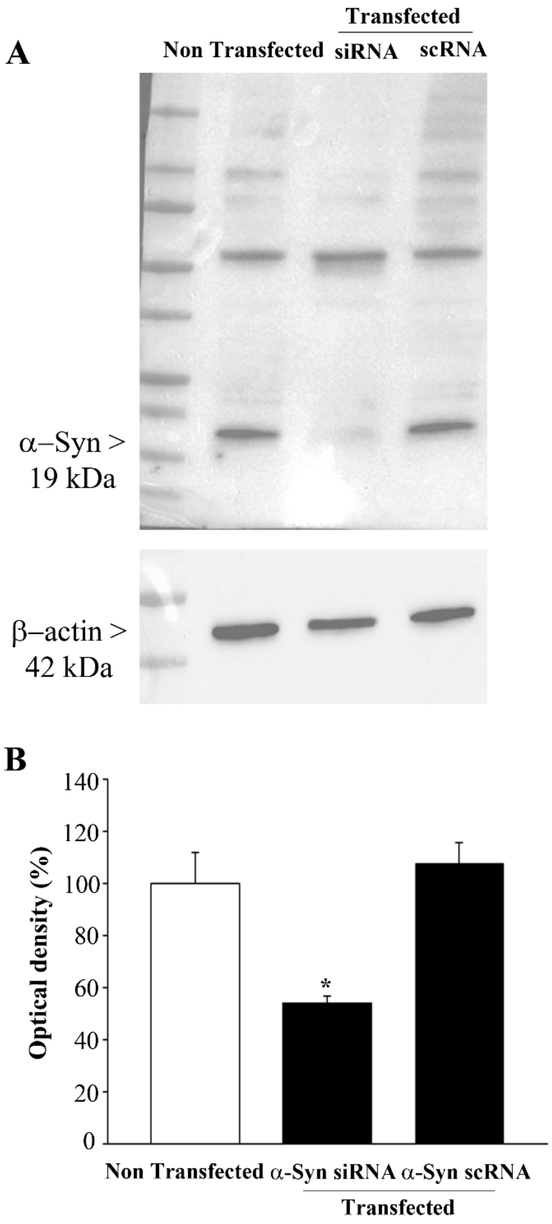

Figure S7. Immuno-blotting for ◎-syn in U87MG cells transfected with ◎-syn siRNA. (**A**) Immunoblotting for ◎-syn in non-transfected, ◎-syn siRNA transfected and scRNA transfected cells is shown. (**B**) The graph reports the optical density as a ratio between the optical density of ◎-syn and that of ◎-actin (non-transfected cells as 100%). Values are given as the mean percentage±S.E.M. of n=3 blots obtained in N = 1 experiment. .

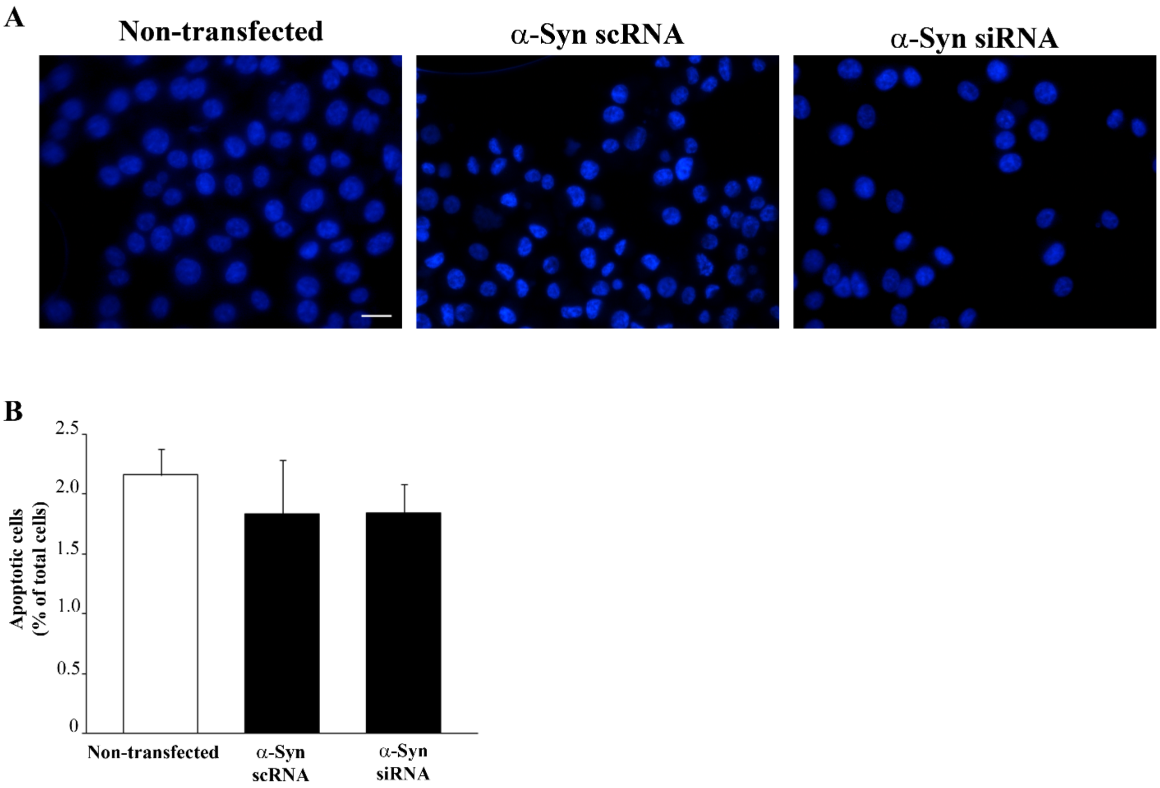

Figure S8. Silencing ⊚-syn in U87MG cells does not induce apoptosis. (**A**) Representative pictures of Hoechst-fluorescent nuclei from non-transfected, scRNA transfected and ⊚-syn siRNA transfected cells are shown. (**B**) Graph reports the percentage of apoptotic cells counted in each experimental group out of the total number of cells. Scale bar = 25 m.
